# Biphasic control of the B cell transcriptome by mTORC1 and GSK3

**DOI:** 10.1016/j.celrep.2025.116361

**Published:** 2025-09-29

**Authors:** Jens Kalchschmidt, Tomoya Kanno, Solji Park, Wendy D. Dubois, Yongbing Zhao, Pawel Trzaskoma, Craig J. Thomas, Louis M. Staudt, John J. O’Shea, Seolkyoung Jung, Rafael Casellas

**Affiliations:** 1 National Institute of Arthritis and Musculoskeletal and Skin Diseases, National Institutes of Health, Bethesda, MD, USA; 2 National Cancer Institute, National Institutes of Health, Bethesda, MD, USA; 3 National Center for Advancing Translational Sciences, National Institutes of Health, Rockville, MD, USA; 4 Present address: Department of Hematopoietic Biology and Malignancy, The University of Texas MD Anderson Cancer Center, Houston, TX, USA; 5 These authors contributed equally; 6 Lead contact

## Abstract

A central question in immune regulation is how cells coordinate transcriptional responses to environmental cues. It remains unclear whether transcriptional regulation is controlled by isolated mechanism or integrated regulatory programs. Here, we develop a high-sensitivity, genome-wide CRISPR-Cas9 screening platform with 47 transcriptional reporters in human B cell lymphoma, identifying 4,440 regulators and 17,638 regulatory interactions. To enable the exploration of these networks, we establish B-LEARN, an interactive portal for data visualization and discovery. Our results reveal a large number of shared regulators across our 47 screens that act as context-dependent activators or repressors. Globally, we uncover a biphasic regulatory architecture in which mTORC1 and GSK3 exert opposing control over the B cell transcriptome. Notably, mTOR inhibition broadly activates key B cell genes, an effect antagonized by GSK3. Thus, B cell transcription is governed by an integrated, pathway-driven circuit, offering new targets to modulate gene expression in lymphoma and autoimmune disease.

## In brief

Kalchschmidt et al. combine 47 reporter cell lines and CRISPR screens to map gene regulation in human B cell lymphoma. They establish the interactive B-LEARN portal and discover that mTORC1 and GSK3 oppositely regulate cell cycle and immune response genes, offering insights and treatment targets for lymphoma and autoimmune diseases.

## Graphical abstract

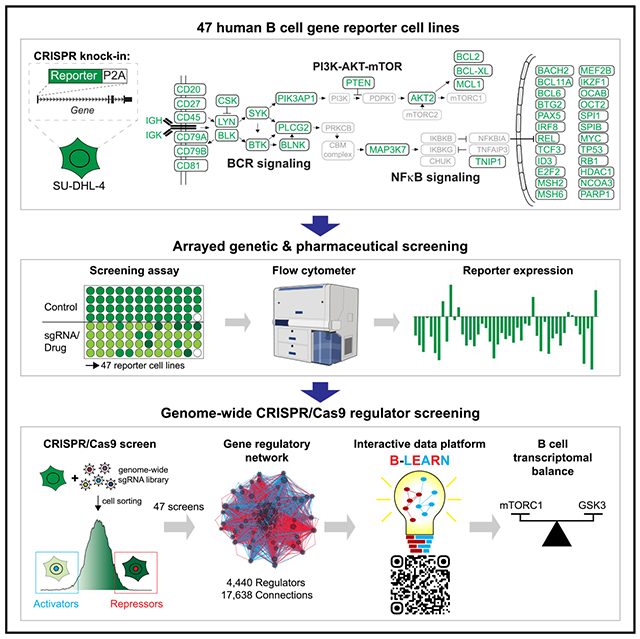

## INTRODUCTION

A central challenge in immune regulation is to understand how immune cells coordinate transcriptional programs in response to metabolic and signaling cues. Although individual transcription factors (TFs) and signaling pathways have been well characterized, it remains unclear to what extent gene expression is governed by discrete, gene-specific mechanisms vs. integrated, higher-order regulatory programs. Deciphering these architectures is essential for understanding how immune cells interpret and respond to environmental stimuli.

B cell activation engages multiple signaling pathways that coordinate dynamic gene expression programs. Following antigen recognition, B cells enter germinal center (GC) reactions, where they undergo affinity maturation and differentiate into antibody-secreting plasma cells or memory B cells.^1^ These processes are tightly regulated to ensure effective and durable adaptive immune responses, as memory B cells and long-lived plasma cells provide sustained protection against reinfection.^2^ Dysregulation of these pathways can lead to immunodeficiency, autoimmunity, or B cell malignancies.^3–5^

Decades of research using animal models and cell lines have uncovered key effectors of B cell function, yet global strategies to dissect the transcriptional regulation of B cell genes have remained limited. CRISPR-based functional genomic screens coupled with fluorescence-activated cell sorting (FACS) have been employed to study the regulation of individual genes in both immune and non-immune cells.^6–10^ To systematically map transcriptional regulation in human B cells, we developed a genome-wide CRISPR-Cas9 screening platform. For this, we engineered 47 transcriptional reporter lines in human diffuse large B cell lymphoma (DLBCL) cells, enabling precise and quantitative measurement of gene expression changes by FACS. Genome-wide loss-of-function screens across these reporters identified 4,440 regulators and 17,638 regulatory interactions spanning key B cell signaling pathways. To facilitate data exploration, we created B-LEARN (B-cell Lymphoma Gene Expression Activator and Repressor Network; https://niams.shinyapps.io/B-LEARN/), an interactive online portal for searching, analyzing, and visualizing transcriptional regulatory interactions.

Unexpectedly, we found that core cellular processes such as ribosome biogenesis and translation acted as context-dependent activators or repressors, collectively accounting for more than 30% of gene regulators in individual screens. Our analysis revealed a complex biphasic regulatory architecture within the B cell transcriptome, shaped by opposing activities of mechanistic target of rapamycin complex 1 (mTORC1) and glycogen synthase kinase 3 (GSK3). Inhibition of mTORC1 with rapamycin suppressed cell cycle and metabolic genes but paradoxically enhanced the expression of immune response and translation-associated genes. Co-inhibition of GSK3 reversed the cell cycle, metabolic, and immune gene programs, uncovering a dynamic regulatory circuit.

These findings demonstrate that B cell transcription is not controlled by isolated pathways but instead is orchestrated through a structured, higher-order regulatory program balanced by opposing activities of mTORC1 and GSK3. By applying high-throughput functional genomics at an unprecedented scale, our work defines a new framework for understanding global transcriptional regulation in B lymphocytes, with broad implications for immunology, cancer biology, and therapeutic development.

## RESULTS

### Human B cell CRISPR-Cas9 gene regulatory screens

**Figure 1.**
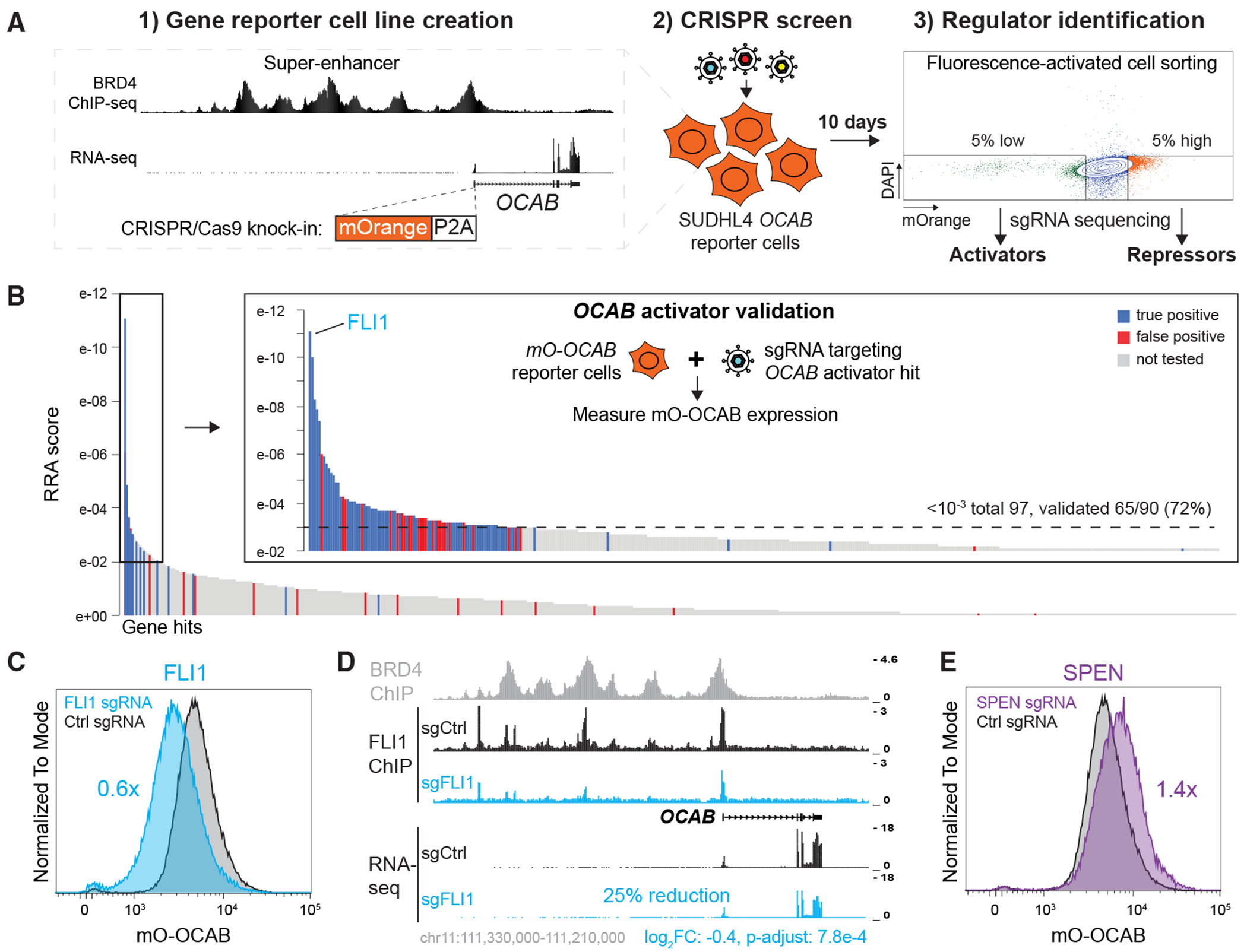
Design and validation of *OCAB* pilot regulatory CRISPR-Cas9 screen (A) Schematic workflow of the CRISPR screen approach. *OCAB* reporter cells are created through *mOrange-P2A* knock-in, transduced with the genome-wide sgRNA library Brunello, and FACS-sorted to identify the activators and repressors of *OCAB* expression. (B) Distribution of *OCAB* activators ranked by the RRA score. Selected activators were individually validated with specific targeting sgRNAs. A total of 113 hits were tested with 74 true positives. (C) mO-OCAB expression measured by flow cytometry 10 days after *FLI1* or control sgRNA transduction. (D) ChIP-seq (FLI1, BRD4) and RNA-seq profiles at the *OCAB* locus in control and FLI1-depleted cells. Fold change and adjusted *p* values (Benjamini-Hochberg) are indicated. (E) As in (C) but after *SPEN* or control sgRNA transduction.

To explore transcriptional regulation in B cells at a global scale, we combined transcriptional reporters with CRISPR-Cas9 genomic screening in the DLBCL line SU-DHL-4.^11^ To optimize the experimental approach, we first labeled the B cell transcriptional coactivator *OCAB* with an mOrange-P2A (mO) reporter (Figure 1A). OCAB is crucial for GC formation, and previous lethality CRISPR screens showed that it is essential for DLBCL viability.^12,13^ To globally identify regulators of *OCAB* expression, knock-in *mO-OCAB* cells were transduced with the sgRNA Brunello library, which targets all human protein-coding genes with up to 4 sgRNAs.^14^ Ten days post-infection, the top and bottom 5% mO^low^ and mO^high^ cells were sorted, and sgRNA enrichment was determined by deep sequencing. Activators and repressors were defined as gene hits identified in the mO^low^ and mO^high^ populations that upon depletion, directly or indirectly, decrease or increase mO-*OCAB* expression, respectively (Figure 1A).

Candidate regulators were ranked by their MAGeCK Robust Rank Aggregation (RRA) scores.^15^ To establish an appropriate RRA score cut-off, we individually validated 163 hits (Figures 1B and S1A). We did this by transducing *mO-OCAB* cells with the most effective sgRNA for each hit and measuring fluorescence via flow cytometry (see STAR Methods). At RRA scores of <10^−3^, we successfully validated 72% of OCAB activators and 97% of repressors (Figures 1B and S1A). Cutoffs either higher or lower than this threshold proved too stringent or overly permissive for downstream analyses.

Interestingly, FLI1 was the highest-ranked *OCAB* activator in our screen (Figure 1B). FLI1 is an ETS-domain TF that plays a key role in hematopoiesis, B cell differentiation and function, and is frequently translocated in human cancer.^16–19^ We confirmed that the depletion of *FLI1* in a bulk population of *mO-OCAB* cells diminished reporter fluorescence (Figure 1C). To determine whether FLI1 directly regulates *OCAB* expression, we performed FLI1 ChIP-seq in SU-DHL-4 cells and observed prominent FLI1 peaks at the *OCAB* promoter and upstream BRD4+ super-enhancer elements (Figure 1D). Importantly, both FLI1 occupancy and *OCAB* expression were reduced upon *FLI1* sgRNA transduction, indicating direct regulation of *OCAB* by FLI1.

On the other hand, we detected SPEN as the highest-ranked *OCAB* repressor (Figure S1A). SPEN (aka SHARP and MINT) is a co-repressor involved in various signaling pathways, essential for X chromosome inactivation, and recurrently mutated in DLBCL.^20–22^ As expected, the depletion of *SPEN* increased mO-OCAB expression, which is consistent with our screening result and SPEN’s prominent repressor function (Figure 1E).

Notably, during our validation, we noticed a cluster of hits on chromosome 11—specifically between the centromere and the *OCAB* locus—whose targeting resulted in a complete loss of mO-OCAB signal in a subset of cells (Figure S1B; e.g., *KLC2*). This is likely caused by Cas9-induced genome instability leading to the deletion of the *mO-OCAB* allele. In contrast, targeting regulators located upstream of the centromere (e.g., *AMBRA1*) or downstream of *OCAB* (e.g., *FLI1*) consistently shifted the mO-OCAB signal in all cells (Figure S1B). To avoid such artifacts, we excluded hits located between the centromere and the knock-in gene locus from further analysis. Together, these findings demonstrate the robustness of our CRISPR-Cas9 screen in reliably identifying the global regulators of gene expression.

### A B cell gene reporter panel for transcriptional regulation and drug discovery

**Figure 2.**
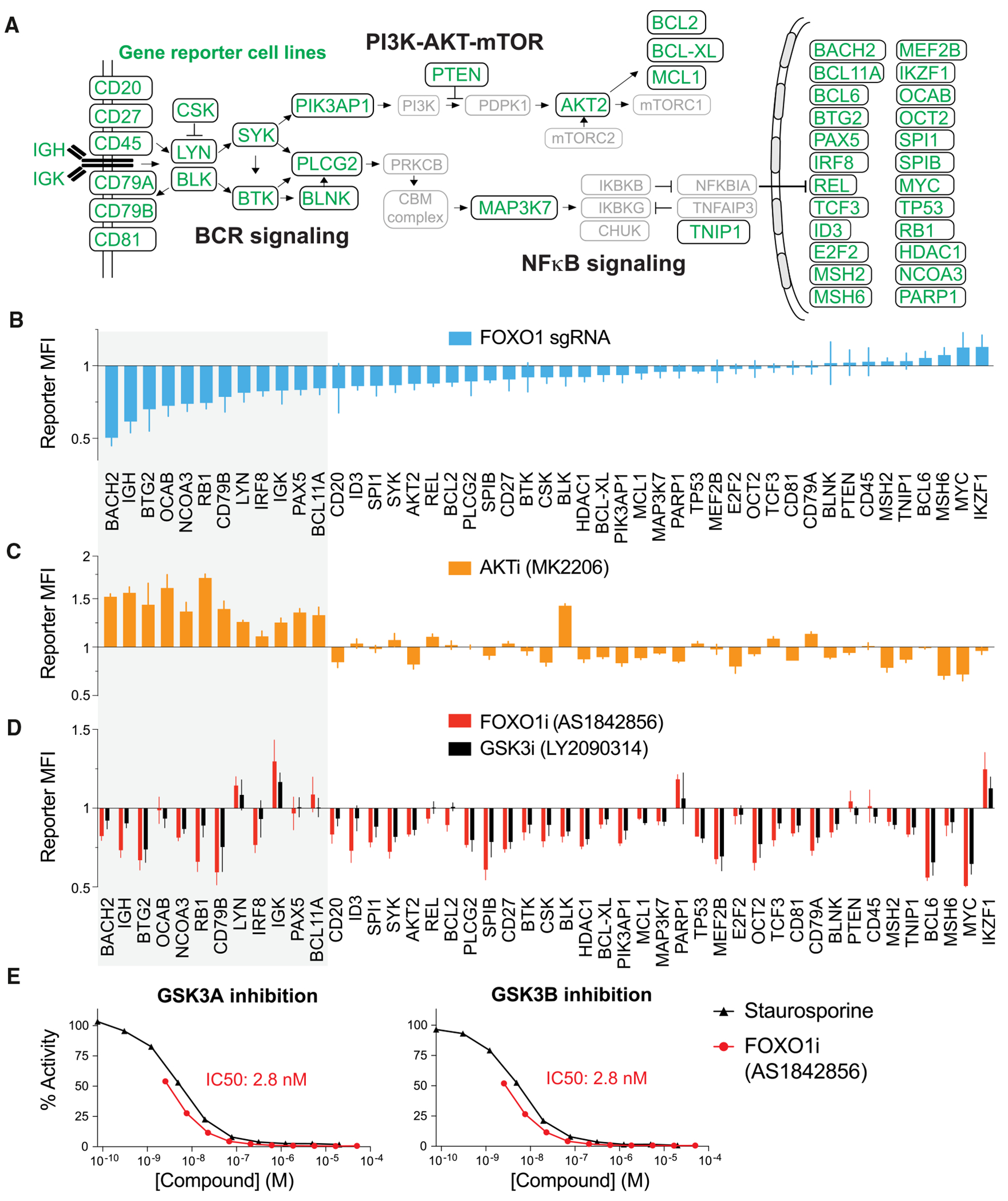
Panel of 47 B cell gene reporter cell lines for genetic and pharmaceutical screening (A) Schematic overview of B cell signaling cascades and selected nuclear proteins. 47 gene reporter B cell lines (highlighted in green) were created. (B–D) Bar graphs showing reporter MFI ± SEM in *FOXO1*-depleted (B), MK2206-treated (C), and AS1842856- or LY2090314-treated (D) cells. (E) *In vitro* GSK3A (left) or GSK3B (right) kinase inhibition using putative FOXO inhibitor (AS1842856) and staurosporine as a control.

To map the regulatory circuitry of human B cells, we generated 46 additional mO or green fluorescent protein (GFP) reporter cell lines by labeling genes critical to B cell function (Figure 2A; Table S1). Targets included key components of BCR signaling, the PI3K-AKT-mTOR and NF-κB pathways, as well as nuclear proteins and TFs such as BCL6, MYC, OCT2, and PAX5. Reporter signal intensities strongly correlated with RNA-seq expression levels (R^2^ = 0.92), confirming that the reporter lines reliably reflect endogenous gene expression (Figure S2A).

To validate the B cell reporter panel, we used CRISPR-Cas9 to deplete *FOXO1* across all 47 reporter cell lines. FOXO1 is a key regulator of bone marrow B cell development, immune tolerance, and GC differentiation.^23–25^ Consistent with these roles, our analysis confirmed FOXO1 as a potent activator of essential B cell genes, including *BACH2, IGH, BTG2*, and *OCAB* (Figure 2B). In parallel, we treated the reporter lines with two compounds known to modulate FOXO1 activity: MK2206, an AKT inhibitor that activates FOXO1, and AS1842856, a commonly used FOXO1 inhibitor.^26^ Consistent with increased FOXO1 activity upon AKT inhibition, MK2206 treatment increased reporter signals for genes most downregulated by *FOXO1* deletion (Figure 2C). In contrast, AS1842856 produced expression profiles that diverged markedly from those observed upon *FOXO1* deletion (Figure 2D), suggesting substantial off-target effects, as recently suggested.^27^

Notably, screening additional small-molecule inhibitors revealed that AS1842856 closely phenocopies the reporter profiles of GSK3 inhibitor LY2090314 (Figure 2D). Indeed, *in vitro* HotSpot kinase assays revealed that AS1842856 potently inhibits GSK3A and GSK3B with an IC50 of 2.8 nM (Figure 2E). Together, these findings validate our reporter cell line panel as a powerful resource for studying gene regulation and for drug target discovery. By enabling direct comparisons between genetic and pharmacological perturbations, this platform facilitates the identification, validation, and refinement of small-molecule target specificity.

### A comprehensive B cell regulatory network

**Figure 3.**
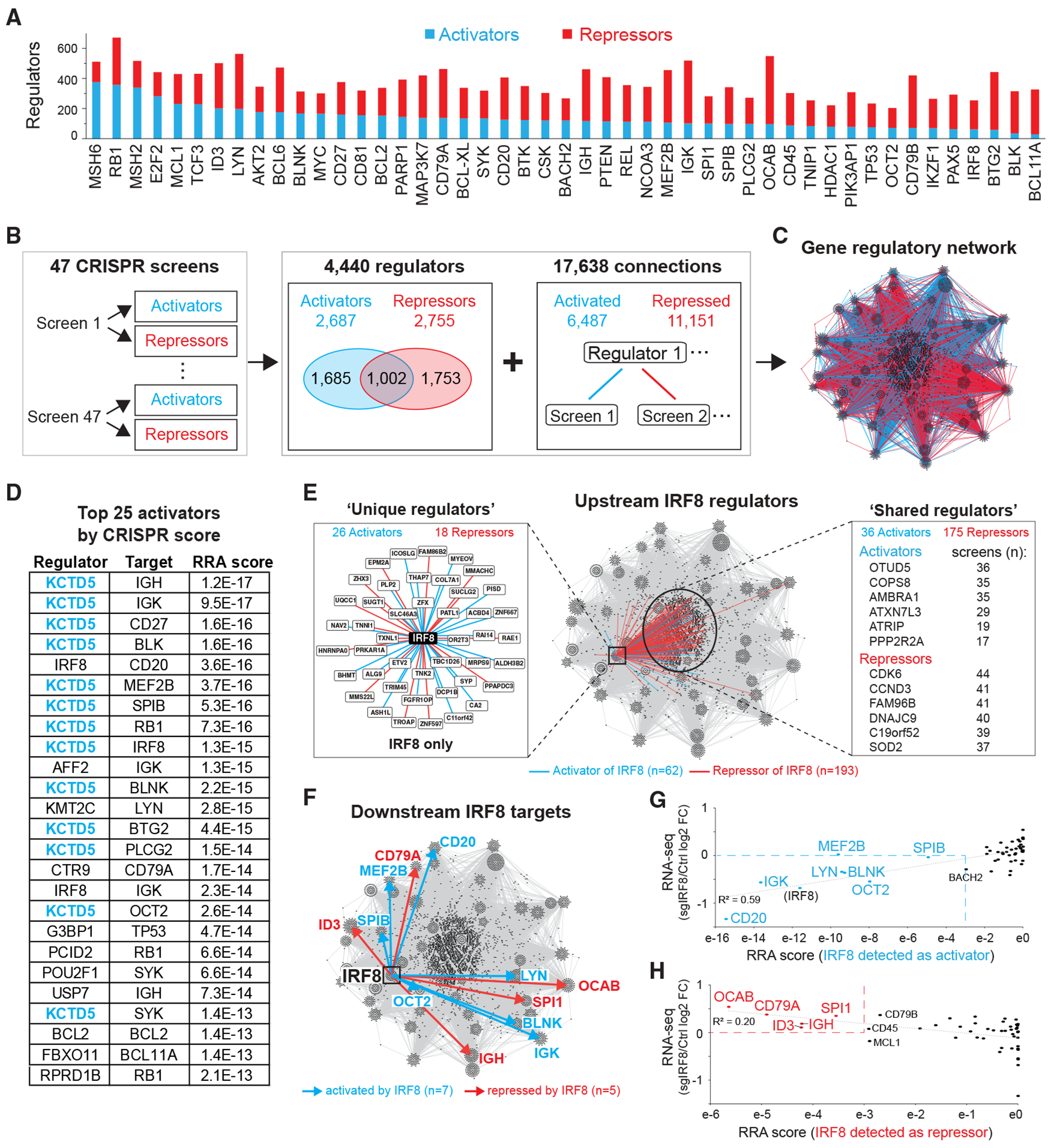
B cell regulatory network generated by 47 CRISPR screens (A) Bar graph shows numbers of activators and repressors detected across all 47 screens. (B) Total numbers of regulators and connections detected in all 47 screens. Venn diagram showing the overlap in regulator function and connections link regulators and target genes. (C) A gene regulatory network was built using Cytoscape, showing all 4,440 regulators and 17,638 connections with direction (activation in blue; repression in red). (D) The top 25 activators ranked by the RRA score and their target genes are shown. *KCTD5*, highlighted in blue, appeared 13 times in the top 25. (E) Upstream IRF8 regulators shown in color are superimposed on the network computed in (C). The left panel shows regulators detected uniquely for IRF8, and the right panel lists select common regulators that impact IRF8 and other genes. (F) Arrows indicate IRF8 downstream target genes superimposed on the network displayed in (C). (G and H) Scatterplots showing correlations between mRNA gene expression changes and CRISPR RRA scores in IRF8-depleted cells for IRF8-activated (G) and -repressed (H) target genes.

To construct a comprehensive gene regulatory network in SU-DHL-4 B cells, we conducted large-scale genome-wide CRISPR screens across all 47 reporter cell lines, as previously done for *OCAB* (Figure 1A; Table S2). For clarity, we refer to sgRNA-enriched hits as “regulators,” identified as activators or repressors, and to the corresponding CRISPR screen as the “target.” On average, each screen identified approximately twice as many repressors (*n* = 237) as activators (*n* = 138), though the ratio varied across the 47 target genes (Figure 3A). For example, *MSH6* was linked to 376 activators and 136 repressors, whereas *BCL11A* was predominantly regulated by repressors (298 repressors vs. 29 activators).

In total, we identified 4,440 regulators, comprising 1,685 activators, 1,753 repressors, and 1,002 context-dependent regulators that acted as activators for some genes and as repressors for others (Figure 3B). The aggregate dataset revealed 17,638 interactions connecting these regulators to the 47 target genes (Tables S3A and S3B). Using these data, we computationally constructed a regulatory network (Figure 3C) incorporating regulator strength (RRA score), functionality (activator vs. repressor), directionality (regulator-to-target), and specificity (unique vs. broadly shared).

Among activators, KCTD5—a ubiquitin E3-ligase substrate adapter protein^28^—consistently ranked highest (Figure 3D). It was also identified as an activator in 32 of the 47 screens, highlighting a significant role in B cell transcriptional regulation (Figures S3A and S3B). Other frequently identified activators included the deubiquitinase OTUD5, the ubiquitin ligase substrate adaptor protein FZR1, and the COP9 signalosome complex subunit COPS8 involved in ubiquitin-mediated processes (Figure S3A). These findings underlie an unexpectedly broad role for the ubiquitin pathway in regulating gene expression in B lymphocytes.

Among repressors, SPEN ranked highest across all 47 screens (Figure S3C), prominently repressing *OCAB* as well as *IGH, REL*, and *PTEN*, among others (Figure S3D). Other frequently identified repressors included CDK6, CCND3, and FAM96B, which are involved in cell cycle regulation and chromosome segregation (Figure S3C).

To explore the B cell regulatory network, we first focused on IRF8, a TF essential at multiple stages of B cell development.^29,30^ Our screen identified 255 IRF8 regulators, which we further classified as either unique to the IRF8 screen or shared across multiple screens (Figure 3E). Unique regulators formed a distinct node centered on IRF8 (Figure 3E, left panel), whereas shared regulators occupied the center of the network (Figure 3E, right panel). Cross-referencing with the Ingenuity Knowledge Base (QIAGEN) confirmed seven previously known regulators, suggesting that the remaining 248 candidates represent potentially unknown IRF8 regulators.

IRF8 itself was detected as a regulator in 12 screens (Figure 3F). Consistent with previous reports,^30–33^ IRF8 activated *CD20, LYN, POU2F2*, and *SPIB*, while repressing *SPI1*. Additionally, our screen revealed uncharacterized IRF8 targets, including *OCAB* and *CD79A*. The reliability of our CRISPR screen in identifying bona fide IRF8 targets was further validated by the correlations between CRISPR RRA scores and RNA-seq data from cells treated with *IRF8*-targeting sgRNA (Figures 3G and 3H). Collectively, these findings underscore the power of our transcriptional regulatory network in uncovering key mechanisms governing B cell transcriptional control.

### B-LEARN, an interactive data portal for exploring the B cell regulator network

**Figure 4.**
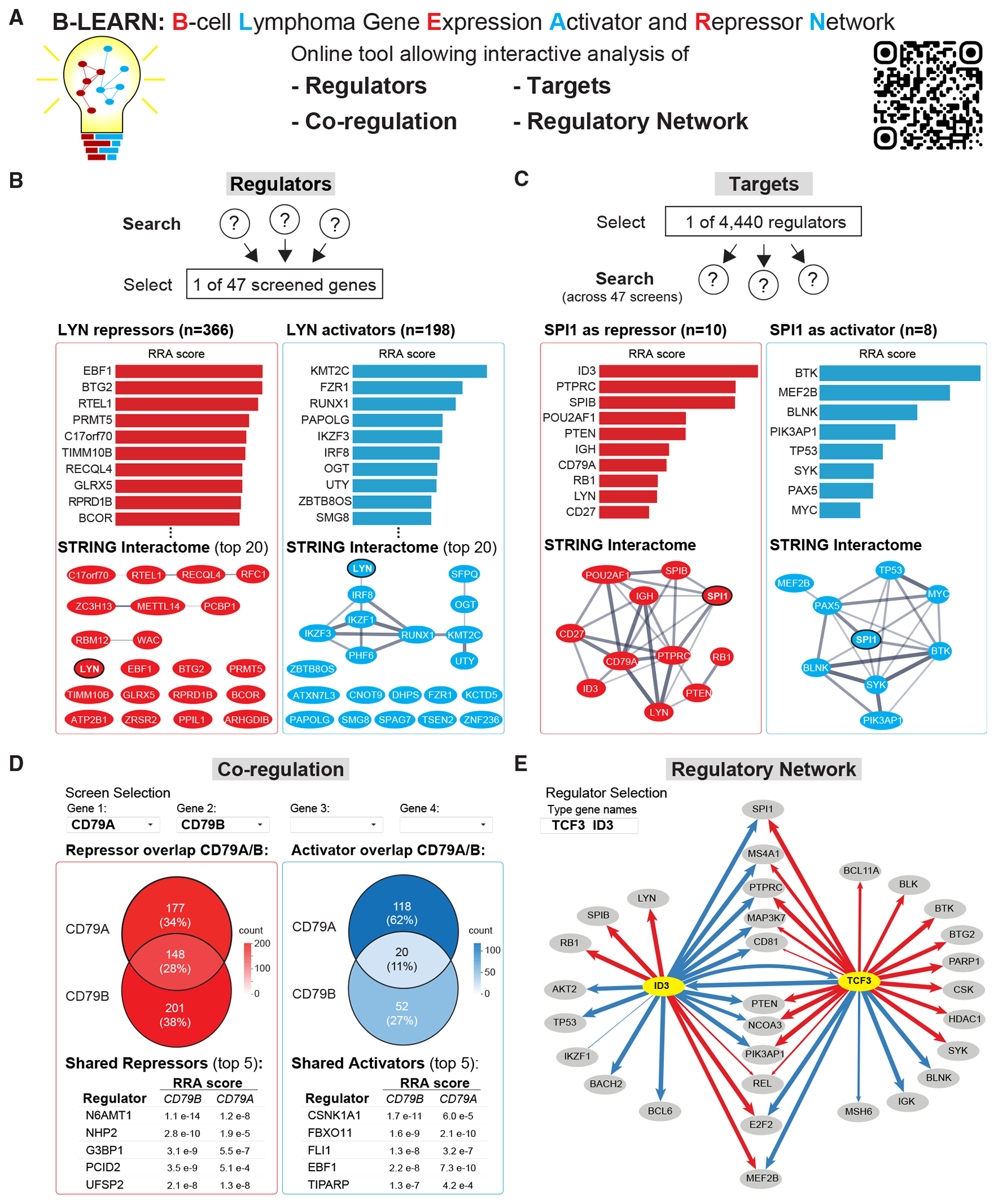
B-LEARN, an interactive data portal to search and visualize regulatory networks (A) B-LEARN (https://niams.shinyapps.io/B-LEARN/) provides four core functionalities. (B) In the “regulators” search, users select one of the 47 CRISPR-screened genes to display upstream regulators. Results are shown as bar graphs for repressors (red) and activators (blue) sorted by RRA scores (low to high), and STRING network visualizations. Regulator search for *LYN* is shown as an example. (C) In the “targets” search, users select any of the 4,440 identified regulators to displays its target genes among the 47 CRISPR screens. Bar graphs and STRING network analyses are shown as in (B). As an example, targets of SPI1 (PU.1) are shown. (D) The “co-regulation” function analyzes regulator overlaps across up to four screens as Venn diagrams and tables. Regulator overlaps between *CD79A* and *CD79B* are shown as an example. (E) The “regulatory network” function allows custom visualization of regulator-target interactions based on our CRISPR screen data. By inputting any of the 4,440 regulators, B-LEARN generates a network where the directionality (arrows from regulator to target), sense (activator in blue or repressor in red), and regulation strength (line width proportional to the RRA score) are displayed. As an example, ID3 and TCF3 (highlighted in yellow) were used as input, and their regulatory network is plotted as output.

To enhance the accessibility and exploration of our data, we developed B-LEARN, an interactive online data portal designed for the intuitive search and visualization of the 4,440 B cell regulators and 17,638 regulatory connections (Figure 4A; https://niams.shinyapps.io/B-LEARN/). The platform features four core functionalities: regulator, target, co-regulation, and regulatory network searches (Figures 4B–4E).

The “regulator” search allows users to explore activators and repressors by selecting any of the 47 CRISPR-screened target genes as input (Figure 4B). Results can be refined using ontology-based filters and by adjusting the cut-off stringency for CRISPR RRA scores. The output includes bar graphs of RRA scores and STRING network visualizations, offering insights into the physical and functional interactions among identified regulators.^34^ Figure 4B presents the B-LEARN regulator analysis of *LYN*, an Src-family kinase that regulates B cell signaling, cancer, and autoimmune disease.^35,36^ Among the top *LYN* activators, we identified the lysine methyltransferase KMT2C and TFs RUNX1, IKZF1/3, and IRF8 (Figure 4B, right). While Runx1 is a known regulator of *Lyn*,^37^ its STRING network associations with IRF8, IKZF1/3, and KMT2C suggest a potential cooperative role in *LYN* activation. On the repressor side, EBF1 and BTG2 emerged as top negative regulators of *LYN* (Figure 4B, left). Notably, neither has been previously implicated in *LYN* repression nor show direct connections to *LYN* in the STRING network.

The “target” search allows users to determine in which of the 47 CRISPR screens a specific regulator was identified. By inputting any of the 4,440 regulator hits, users can retrieve their associated activated or repressed target genes (Figure 4C). For instance, the TF PU.1 (encoded by *SPI1*) was identified as an activator of *BTK, BLNK, SYK*, and *PAX5*, aligning with previous studies.^38–42^ Additionally, we identified *MEF2B* as a PU.1-activated target. On the repressor side, PU.1 was found to negatively regulate the inhibitor of DNA binding 3 (*ID3*), CD45 (*PTPRC*), and SPI-B, expanding its known repressive roles.

The “co-regulation” search analyzes regulator overlaps across all screens, providing insights into broader co-regulatory mechanisms. Users can query up to four genes simultaneously to identify shared activators and repressors (Figure 4D). For example, this analysis identified 20 shared activators and 148 shared repressors between *CD79A* and *CD79B*, the immunoglobulin-associated α- and β-chains essential for BCR signaling (Figure 4D). Among these, well-established *CD79A/B* transcriptional activators include EBF1 and FLI1.^43–45^ Additionally, frequently detected activators (e.g., casein kinase 1 [*CSNK1A1*]) and repressors (e.g., *N*-6 adenine-specific DNA methyltransferase [*N6AMT1*]) likely contribute to broader transcriptional control mechanisms, warranting further investigation below.

The “regulatory network” function enables custom visualization of complex interactions between regulators and their targets. By inputting regulator names, users can generate a network based on our CRISPR screen data, where the sense (activator in blue or repressor in red), directionality (arrows from regulator to target), and regulation strength (line width proportional to RRA score) are displayed. As an example, we examined the regulatory network of E2A (encoded by *TCF3*) and its known negative regulator ID3 (Figure 4E). Our analysis identified TCF3 as the top-ranked activator of *ID3*, consistent with previous studies.^46,47^ However, we also identified a reciprocal activation from ID3 to *TCF3*, suggesting a positive feedback loop. Overall, we detected 20 ID3 and 23 TCF3 targets, with many genes co-regulated in opposing directions, as indicated by the blue and red arrows in the network. This pattern aligns with ID3’s role in inhibiting E protein DNA binding, thereby counteracting TCF3 transcriptional activity. Interestingly, TCF3 exhibited a predominantly repressive role within this subset of target genes.

In summary, the B-LEARN portal provides intuitive, user-friendly access to extensive datasets, enabling custom exploration and visualization of previously unrecognized gene regulatory interactions and mechanisms.

### Broad bi-phasic impact of mTOR signaling

**Figure 5.**
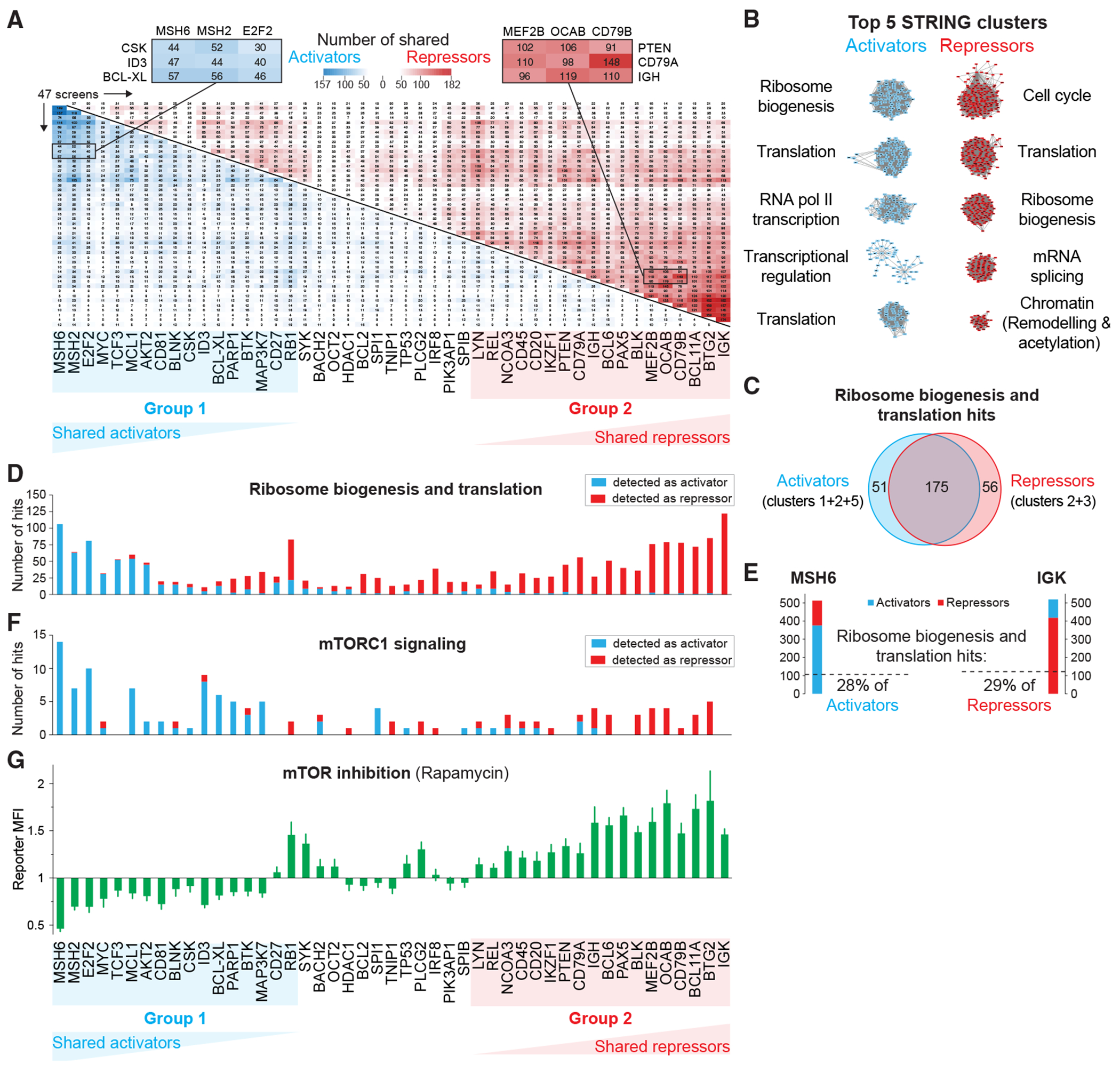
Global CRISPR screen regulator analysis identifies mTOR as master regulator (A) Co-regulation analysis was performed to identify shared regulators among the 47 CRISPR screens. The symmetric matrix displays activator (blue) and repressor (red) overlaps across all combinations of gene pairs. Genes are arranged according to regulator overlap (see Figures S4A–S4C). Close-up panels show selected activator and repressor overlaps. (B) The top 5 STRING-MCL clusters are shown separately for activators and repressors. (C) Venn diagram showing the overlap of regulators detected as activators or repressors in the ribosome biogenesis and translation clusters. (D) Activator and repressor counts shown in (C) are graphed per screen across all 47 genes (same order for A, D, F, and G). (E) The fractions of regulators belonging to ribosome biogenesis and translation clusters are shown for MSH6 activators (left) and IGK repressors (right). (F) Bar graph displays the count distribution of hits involved in mTORC1 signaling that were detected as activators or repressors in the 47 screens. (G) Bar graph showing relative reporter MFI ± SEM of 2-day rapamycin vs. DMSO-treated cells.

To reveal global patterns of transcriptional regulation, we identified shared regulators across all screened gene pairs and applied hierarchical clustering for visualization (Figures 5A and S4A–S4C). Notably, this approach uncovered two distinct gene groups in B cells. Group 1 (17 genes) exhibited a high degree of shared activators (Figure 5A, lower blue matrix). For instance, *BCL-XL* shared 57, 56, and 46 activators with *MSH6, MSH2*, and *E2F2*, respectively (Figure 5A, left zoomed-in panel), accounting for 34%–42% of all its activators. Group 2 (18 genes) displayed a high degree of shared repressors (Figure 5A, top red matrix). For example, *CD79A* shared 110, 98, and 148 repressors with *MEF2B, OCAB*, and *CD79B*, respectively (Figure 5A, right zoomed-in panel), comprising 30%–46% of all *CD79A* repressors.

The surprisingly high degree of shared activators and repressors between the two gene groups suggests that B lymphocytes regulate their transcriptome through a limited set of overarching pathways. To investigate this further, we applied STRING analysis combined with Markov clustering to categorize all activators and repressors based on their biological functions (Figure 5B; Tables S4A and S4B). As expected, the top activator clusters included key regulators of transcription and translation, including RNA polymerase II, the mediator and integrator complexes, and tRNA synthases (Figures 5B and S4D).

However, among the top STRING clusters, ribosome biogenesis and translation were found to function as both activators and repressors (Figure 5B). This was unexpected, as disrupting protein translation would typically be expected to reduce, rather than increase, reporter expression. Crucially, this dual classification was not due to different factors within each cluster exerting opposing effects. Instead, we found that 175 out of 282 ribosome regulators (62%) functioned as both activators and repressors (Figure 5C). Consistent with this view, when we examined the distribution of ribosome and translation-related hits, we observed a clear pattern: group 1 genes were preferentially activated by ribosome/translation regulators, while group 2 genes were predominantly repressed by the same factors (Figure 5D). Notably, ribosome/translation-linked regulators accounted for approximately 30% of all activators or repressors detected for *MSH6* (group 1) and *IGK* (group 2), respectively (Figure 5E). In contrast, other top activator and repressor clusters displayed a more uniform regulatory distribution (Figure S4E).

To identify the molecular mechanisms underlying the differential distribution of group 1 and group 2 genes, we searched for regulator clusters capable of recapitulating their partitioning. Our analysis identified energy metabolism proteins, tRNA synthases, and most notably, mTORC1 signaling components as key factors fulfilling this criterion (Figure 5F). The serine/threonine kinase mTOR is a central regulator of cellular growth, integrating nutrient availability, energy status, and environmental cues to control metabolism, protein synthesis, proliferation, and autophagy.^48^ mTOR plays critical roles in regulating immune responses, and its activity generally increases upon immune cell stimulation.^49^ Recent screens of mTORC1 regulators showed that perturbation of mitochondrial function or tRNA synthases leads to lower mTORC1 activity^50,51^—consistent with our findings that factors from these clusters function as activators for group 1 and repressors for group 2 genes. Based on this, we hypothesized that mTORC1 inhibition should replicate the partitioning observed when targeting ribosome biogenesis and translation regulators. Indeed, treatment with rapamycin, a potent mTORC1 inhibitor, led to reduced expression of group 1 genes while increasing the expression of group 2 genes, closely mirroring our CRISPR screening results (Figure 5G). Interestingly, while downregulation by rapamycin was evident within 24 h, upregulation of group 2 genes emerged only after 48 h, suggesting a delayed compensatory response (Figure S4F).

In summary, our analysis uncovered a striking global pattern in B cell transcriptional regulation, identifying two distinct gene groups that exhibit opposing expression changes upon perturbation of essential biological processes. Most notably, we identified mTORC1 as a master regulator behind this transcriptional response. The regulation is both directional—activating group 1 genes while repressing group 2 genes—and temporal, with downregulation occurring rapidly, whereas upregulation follows a delayed, compensatory trajectory.

### mTOR suppresses immune response genes

**Figure 6.**
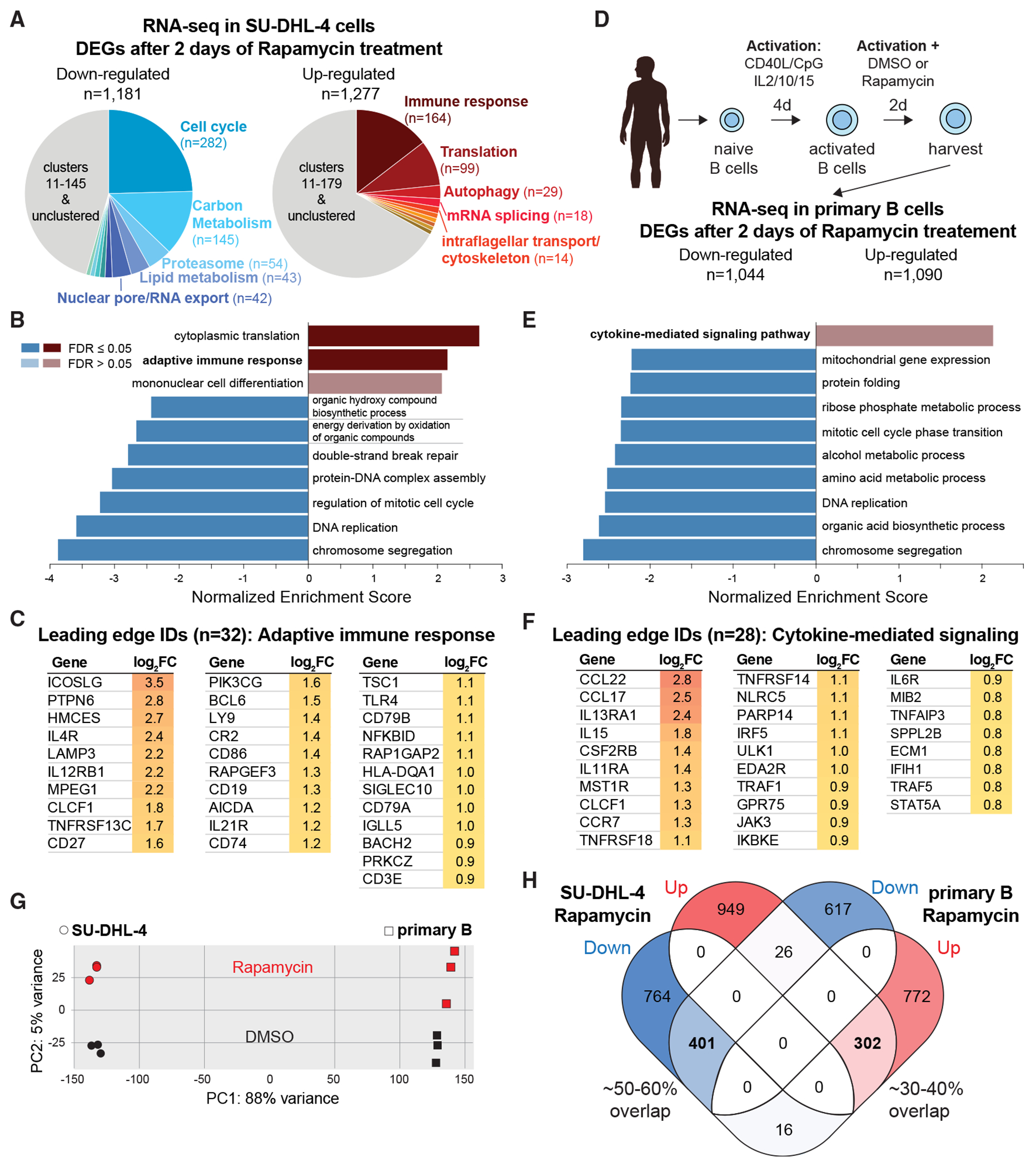
mTOR inhibition induces immune genes in SU-DHL-4 and primary B cells (A) DEGs in rapamycin-treated SU-DHL-4 cells were analyzed with STRING and MCL clustered. Pie charts represent cluster sizes, and functional enrichment for the top 5 clusters is denoted. (B) Gene set enrichment analysis for rapamycin-treated SU-DHL-4 cells. False discovery rates (FDR) based on permutation testing are indicated. (C) List of leading-edge genes from the “adaptive immune response” gene set detected in (B). (D) Schematic experimental overview to assess the transcriptome of rapamycin-treated human primary B cells. Illustration from the NIAID NIH BIOART source (bioart.niaid.nih.gov/bioart/238). (E) Gene set enrichment analysis for rapamycin-treated primary B cells. (F) List of leading-edge genes from the “cytokine-mediated signaling pathway” detected in (E). (G) Principal component analysis of RNA-seq samples derived from rapamycin- or DMSO-treated SU-DHL-4 and primary B cells. (H) Venn diagram showing the overlap between significantly down- or upregulated genes in rapamycin-treated SU-DHL-4 and primary B cells.

To further investigate the effect of mTORC1 inhibition on the B cell transcriptome, we performed RNA-seq on 2-day rapamycin-treated SU-DHL-4 cells (Figure S5A). We identified 2,458 DEGs, with similar numbers of upregulated and downregulated genes (Table S5). As expected, mTORC1 inhibition impaired cell growth, and STRING analysis revealed the two most significantly downregulated gene clusters as cell cycle regulation and metabolism (Figure 6A, left; Table S6A). Notably, the expression of *MSH2*, *MSH6*, and *E2F2* was significantly decreased, consistent with our reporter cell lines (Figure 5G).

Surprisingly, 21 of the 47 CRISPR-screened genes—primarily group 2 genes—were among the most significantly upregulated (Figure 6A, right; Table S6B), further corroborating our screening results. Many of these genes belong to the largest upregulated cluster, which is associated with immune response activation—a paradoxical finding given that rapamycin is used as an immunosuppressant.

To validate this observation, we performed a gene set enrichment analysis (GSEA). Consistent with the STRING results, GSEA revealed the downregulation of chromosome segregation and metabolic pathways, alongside an increase in cytoplasmic translation and adaptive immune response genes (Figures 6B and S5B). Notably, the upregulated immune genes included key activation molecules (e.g., *IL4R* and *CD86*), components of the BCR complex (e.g., *CD79A* and *CD79B*), TFs (e.g., *BACH2* and *BCL6*), and activation-induced cytidine deaminase (*AICDA*; Figure 6C). Proteomic profiling confirmed that rapamycin-mediated transcriptional changes were reflected at the protein level (Figures S5C and S5D; Tables S7A and S7B). These findings thus position mTORC1 as a central regulator of key genes in B cells, highlighting its unexpected role in suppressing immune activation.

Since SU-DHL-4 is a B cell lymphoma line, we questioned whether primary human B cells would exhibit a similar response to mTORC1 inhibition. To test this, we activated naive B cells from three healthy donors and treated them for 2 days with rapamycin or DMSO, followed by transcriptome analysis (Figures 6D and S5E; Table S8). Compared to SU-DHL-4, we observed a similar number of up- and downregulated DEGs (Figures 6A and 6D). As in SU-DHL-4, GSEA analysis revealed the downregulation of chromosome segregation and metabolic processes, alongside the upregulation of cytokine-mediated signaling pathways (Figures 6E and S5F). Notably, a closer inspection of the upregulated immune response genes identified key cytokines and chemokines (e.g., *CCL22* and *IL15*), receptors (e.g., *IL13RA1* and *IL11RA*), signaling molecules (e.g., *JAK3*), and TFs (e.g., *STAT5A*; Figure 6F). Overall, the rapamycin-induced transcriptional response in primary B cells closely mirrored that of SU-DHL-4, indicating a conserved function of mTORC1 in both normal and malignant B cells.

To directly compare the transcriptional response of SU-DHL-4 and primary B cells, we performed principal component analysis (PCA) on RNA-seq data from rapamycin- and DMSO-treated cells (Figure 6G). While the two cell types exhibited distinct separation along PC1, their response to rapamycin was strikingly similar, as reflected in PC2. Furthermore, differential expression analysis revealed a strong overlap of DEGs that were regulated in the same direction in response to rapamycin across both cell types (Figure 6H). Taken together, these findings confirm our CRISPR screen results and demonstrate that 2-day mTOR inhibition unexpectedly enhances immune response gene expression in both lymphoma and primary B cells.

### GSK3 counteracts mTOR for immune response but not for translation control

**Figure 7.**
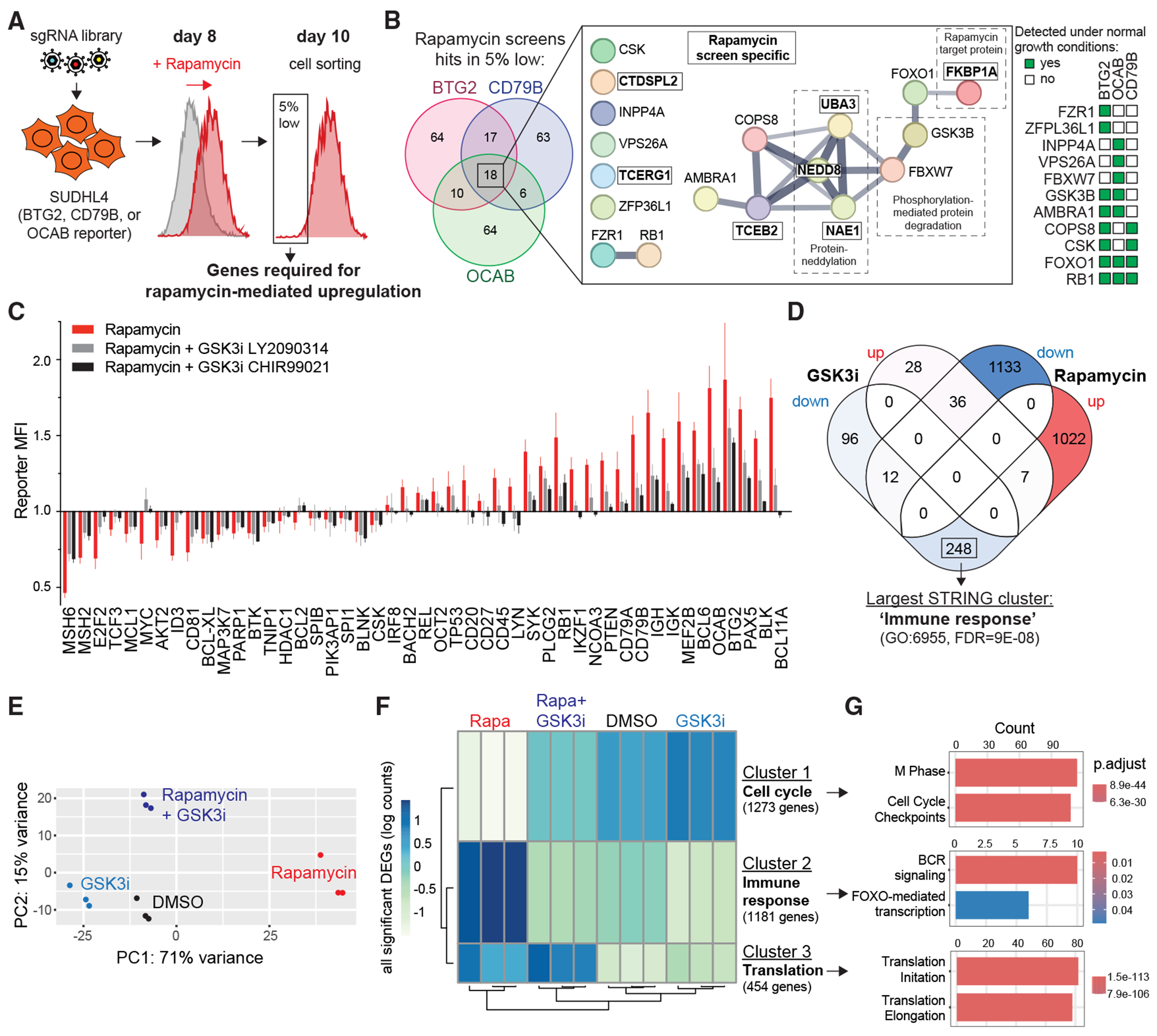
mTOR and GSK3 opposingly regulate cell cycle and immune response gene expression (A) Schematic experimental layout to identify regulators required for rapamycin-mediated induction of BTG2, CD79B, and OCAB. (B) Venn diagram showing the regulator overlap among the 3 rapamycin-treated screens (left). STRING network analysis of the 18 regulators detected in all three screens (middle). Regulators identified specifically in the rapamycin-treated conditions, but not in normal conditions, are boxed and highlighted in bold. The table on the right indicates the screens in which regulators were detected under normal conditions. (C) Bar graph showing relative reporter MFI ± SEM of inhibitor treatments (rapamycin alone or combined with 2 different GSK3 inhibitors) vs. DMSO. (D) Venn diagram showing the DEG overlaps of down- and upregulated genes comparing GSK3 inhibitor-treated vs. rapamycin-treated SU-DHL-4 cells. STRING functional enrichment was performed for genes reduced by GSK3 inhibition but increased by rapamycin. Gene Ontology term and false discovery rates (Benjamini-Hochberg) are indicated for the largest STRING cluster. (E) RNA-seq PCA analysis of SU-DHL-4 cells treated with inhibitors or DMSO. (F) Gene expression heatmap and hierarchical clustering of all differentially expressed genes derived from indicated inhibitor- or DMSO-treated SU-DHL-4. (G) Reactome pathway enrichment analysis of genes in the three main clusters detected in (F). Gene counts and adjusted *p* values (Benjamini-Hochberg) are indicated.

To investigate the mechanism underlying the unexpected induction of immune response genes upon mTORC1 inhibition, we conducted CRISPR-Cas9 screens in the presence of rapamycin using three reporter cell lines (*BTG2*, *CD79B*, and *OCAB*), whose expression was previously shown to increase following rapamycin treatment (Figure 5G). We hypothesized that genes required for rapamycin-mediated upregulation would be enriched in the low-fluorescence population upon deletion (Figure 7A). Indeed, our screens identified 18 common factors across all three rapamycin CRISPR screens (Figure 7B), strongly supporting their role in mediating this response. The top regulator was *FKBP1A*, which encodes FKBP12—a critical mediator of rapamycin-induced mTORC1 inhibition^52^—serving as an internal control for our screens.

Moreover, six additional regulators (*NAE1*, *UBA3*, *NEDD8*, *TCEB2*, *CTDSPL2*, and *TCERG1*)—which were not detected under normal growth conditions—were identified with high confidence in all three rapamycin screens, suggesting a shared role in the response to mTORC1 inhibition (Figure 7B). Among these, NAE1 and UBA3 are required to transfer NEDD8 onto target proteins, e.g., Cullin-RING E3 ubiquitin ligases, which require neddylation for full activation.^53^ Consistent with this, our screen also identified two ubiquitin E3 ligase components TCEB2 and FBXW7 (Figure 7B).^54,55^ FBXW7 is a well-established regulator of oncoprotein degradation, targeting proteins such as cyclin E, MYC, and MCL1, which must first be phosphorylated by GSK3 or other kinases before degradation. In line with this mechanism, we observed reduced MYC and MCL1 reporter levels upon rapamycin treatment (Figure 5G) and detected GSK3B in all three rapamycin screens (Figure 7B), suggesting that GSK3 plays a critical role in the cellular response to mTORC1 inhibition.

To determine whether GSK3 kinase inhibition could counteract the effects of rapamycin, we treated cells with the GSK3 inhibitor LY2090314 (GSK3i). Co-treatment with rapamycin and GSK3i effectively rescued cell proliferation, maintained regular cell size, and prevented G1 cell-cycle arrest (Figures S6A–S6D), consistent with previous findings.^56^ Crucially, we found that co-treatment with rapamycin and two different GSK3 inhibitors impaired rapamycin-induced expression changes in both group 1 and group 2 genes (Figure 7C). This effect required the inhibition of both GSK3A and GSK3B, as only sgRNA-mediated co-depletion—but not individual targeting—was sufficient to counteract the rapamycin response (Figure S6E).

We next evaluated the global transcriptomic effects of GSK3 inhibition and identified 427 DEGs, the majority of which (*n* = 356, or 83%) were downregulated (Table S9). To further explore the connection between GSK3 and mTOR, we directly compared DEGs from GSK3i- and rapamycin-treated SU-DHL-4 cells (Figure 7D). Strikingly, we found that 70% of genes downregulated by GSK3 inhibition (*n* = 248) were upregulated upon rapamycin-mediated mTOR inhibition, suggesting a reciprocal regulatory relationship between these pathways. Further analysis of these reciprocally regulated genes revealed a strong functional enrichment for immune response pathways, highlighting a key role for GSK3 in modulating immune gene expression downstream of mTORC1 signaling (Figure 7D).

These findings prompted us to compare the global transcriptomic profiles of cells treated with GSK3i, rapamycin, or their combination, alongside DMSO controls (Table S10). PCA analysis revealed that PC1 distinctly separated GSK3i- and rapamycin-treated cells from controls but in opposite directions, confirming that GSK3 and mTOR exert counteracting roles (Figure 7E). Further supporting this idea, co-treatment with GSK3i and rapamycin reversed the principal effect of rapamycin treatment along PC1, restoring transcriptomic balance, while maintaining a distinct PC2 profile compared to DMSO controls (Figure 7E).

To further dissect the dual inhibition of GSK3 and mTOR, we clustered all DEGs across the four experimental conditions. This analysis revealed that the rapamycin-induced downregulation of cell cycle genes (cluster 1) and upregulation of immune response genes (cluster 2) were GSK3 dependent, as their expression levels were restored to control upon GSK3i co-treatment (Figures 7F and 7G). However, the rapamycin-mediated increase in translation factor expression (cluster 3) was independent of GSK3, as these genes remained elevated compared to controls, likely explaining the distinct PC2 profile upon GSK3i and rapamycin co-treatment (Figures 7E, 7F, and 7G). Thus, while rapamycin has both GSK3-dependent and GSK3-independent effects, our analysis uncovered a broad set of cell cycle and immune response genes that are oppositely regulated by mTORC1 and GSK3. This suggests that GSK3 plays a crucial role in maintaining transcriptional balance and that perturbation of either pathway has the potential to dysregulate immune responses.

## DISCUSSION

Decades of research have sought to identify key regulators of transcriptional control in immune cells, yet most studies have focused on individual genes in isolation. While valuable, this approach limits our ability to discern global patterns orchestrating the transcriptome. A systems-level analysis of gene regulation can enhance our understanding of disease mechanisms and identify novel therapeutic targets for immune cell-associated disorders, including cancer and autoimmunity.

Here, we present B-LEARN, an interactive data portal built upon 47 CRISPR-Cas9 screens, encompassing 4,440 regulators and 17,638 regulatory interactions in B cells. This platform enabled us to explore gene regulatory networks at an unprecedented scale, facilitating co-regulation analysis, target identification, and visualization of complex transcriptional relationships. We detected previously known regulatory connections such as IRF8-mediated activation of *CD20*, *LYN*, *POU2F2*, and *SPIB* but repression of *SPI1*, which validates our datasets.^30–33^ In addition, we discovered many uncharacterized candidates, such as FLI1 and SPEN as positive and negative regulators of *OCAB*, respectively. All three are frequently dysregulated in cancer, meriting further investigations into potential causal connections.

Alongside B-LEARN, the accompanying panel of gene reporter cell lines provides a powerful platform for high-throughput, fluorescence-based screening. As a proof of principle, we demonstrated that this system can identify genuine FOXO1 target genes and uncover off-target effects, e.g., GSK3 inhibition by the FOXO1 inhibitor AS1842856, which was recently independently reported.^57^ This underscores the strength of our comparative fluorescence profiling approach in detecting unexpected drug-target interactions, making it a valuable tool for small-molecule target screening and mechanistic studies.

To date, regulatory CRISPR screens in B cells have largely focused on individual genes, such as *CD19*, *CD40*, and *IRF4*.^58–63^ While these studies have identified gene-specific regulators, they cannot reveal broader co-regulatory mechanisms governing transcriptional responses across multiple genes. By integrating 47 CRISPR screens, our co-regulation analysis uncovered two major gene groups controlled by a shared set of factors—group 1 being transcriptionally activated, and group 2 repressed by the same regulators. Strikingly, these regulators were highly enriched for components of ribosomal translation, and mTORC1 signaling accounting for a substantial proportion (up to 30–40%) of hits in individual screens. This likely explains the consistent identification of KCTD5, a known modulator of AKT-PI3K-mTOR signaling,^64^ as a top-ranked activator. mTOR signaling is essential for B cell development, GC reactions, and antibody responses.^65–72^ mTORC1 activity in GC B cells is dynamically regulated and has to be tightly controlled, as both hyperactivation and insufficiency impair GC function with reduced levels of BCL6 and AID expression.^65,69,71,72^ Supporting mTORC1’s central role, a recent CRISPR screen in Burkitt’s lymphoma identified mitochondrial one-carbon metabolism as a regulator of mTORC1-dependent autophagic degradation of TCF3,^73^ a finding consistent with our TCF3 reporter downregulation upon rapamycin treatment.

Using our dataset, we made the unexpected observation that prolonged mTORC1 inhibition with rapamycin led to increased immune gene expression, a paradox given that rapamycin is used as an immunosuppressant in organ transplantation and autoimmune disease. While previous studies have reported context-dependent immunostimulatory effects of mTOR inhibition,^74^ these findings have typically been limited to phenotypic assays or focused on a few inflammatory markers.^75–81^ Similar to our observation, rapamycin-treated activated human B cells displayed an activated phenotype with increased numbers of CD23-, CD25-, and CD69-expressing cells.^81^ Our study expands this by providing a genome-wide transcriptomic perspective in both lymphoma and primary B cells, revealing the extent and scale of immune gene upregulation upon mTOR inhibition.

A key discovery is that GSK3 functions as a critical co-regulator of the immune response, opposing the effects of mTORC1 inhibition. While this is consistent with earlier reports studying individual markers,^79,82,83^ our analysis identified GSK3 as an essential global effector that counterbalances mTORC1 activity, activating immune gene expression, while mTORC1 suppresses it. This uncovers a previously unrecognized transcriptional balance between mTORC1 and GSK3, linking fundamental metabolic and signaling pathways to the control of cell cycle progression and immune responses. Future work should aim to identify the downstream effectors driving this transcriptional shift. A prime candidate was cMYC, as it is translationally induced through mTORC1 and targeted for degradation via GSK3 phosphorylation.^84,85^ However overexpression of a degradation-resistant cMYC mutant failed to rescue rapamycin-induced reporter changes (data not shown). Identifying the precise mechanism could have broad implications for diseases treated with mTOR inhibitors (e.g., cancer, autoimmune disorders, and transplantation) and emerging GSK3-targeted therapies (e.g., Alzheimer’s, diabetes, and bipolar disorder). Understanding this mTORC1-GSK3 transcriptional axis may guide therapeutic strategies to fine-tune immune responses in both health and disease.

### Limitations of the study

Our study has several limitations. First, the number of genes and cell lines examined was constrained by the substantial time and effort required to generate reporter cell lines and conduct CRISPR screens. In this study, all findings are based on a GC-derived lymphoma cell line. Due to the nature of this model, the results presented here are unlikely to capture the entire transcriptional regulation involved in various B cell cancers or primary B cells. Future studies should include additional genes, diverse cell line models, and primary cells to assess the generalizability of our findings and determine their biological role, e.g., during B cell differentiation. Second, the validation of *OCAB* (Figures 1B and S1A) revealed the presence of false-positive hits in our datasets. While applying more stringent RRA scores thresholds can reduce these, it also limits the number of candidate regulators available for downstream analysis. In B-LEARN, users can select RRA thresholds appropriate to their specific research needs. Lastly, our current CRISPR screening approach cannot distinguish between direct and indirect gene regulation. Disentangling these effects will require complementary methods such as ChIP-seq and RNA-seq in regulator-depleted cells, as demonstrated for FLI1 as top activator of *OCAB* (Figure 1D). We suspect that many of the 4,440 identified regulators influence gene expression indirectly by perturbing broader pathways—such as mTORC1 signaling. Uncovering these complex mechanism underscores the value of integrating results across multiple CRISPR screens to build large-scale regulatory networks.

## RESOURCE AVAILABILITY

### Lead contact

Further information and requests for resources and reagents should be directed to and will be fulfilled by the lead contact, John J. O’Shea (john.oshea@nih.gov).

### Materials availability

The 47 SU-DHL-4 reporter cell lines generated in this study have been deposited to ATCC and are publicly available.

### Data and code availability

- Deep-sequencing data generated in this study have been deposited at GEO and are publicly available as of the date of publication. The proteomics data have been deposited to the ProteomeXchange Consortium via the PRIDE^86^ partner repository and are publicly available as of the date of publication. The accession numbers are listed in the key resources table.
- The source code developed for B-LEARN has been deposited at Zenodo and is publicly available as of the date of publication. The accession number is listed in the key resources table.
- Any additional information required to reanalyze the data reported in this paper is available from the lead contact upon request.

## STAR★METHODS

### EXPERIMENTAL MODEL AND STUDY PARTICIPANT DETAILS

#### Cell lines

SU-DHL-4 is a lymphoblastic cell line isolated from the peritoneal effusion of a White, 38-year-old, male patient. SU-DHL-4 cells with doxycycline inducible Cas9 were described previously.^13^ Cells were authenticated by Sanger sequencing to carry a previously reported t(14;18) translocation between BCL2 and the immunoglobulin heavy chain locus (*BCL2*:*IGH*).^95^ Cells were cultured in advanced RPMI-1640 media supplemented with 5% FBS (Gemini), 1% GlutaMAX, and 1% Penicillin-Streptomycin (Thermo Fisher Scientific).

HEK293T cells were grown in DMEM media supplemented with 10% FBS (Gemini), 1% GlutaMAX, and 1% Sodium Pyruvate (Thermo Fisher Scientific).

Cells were tested routinely for mycoplasma contamination.

#### Primary human B cells

Acquisition of blood from human volunteers and informed consent is obtained through the standard National Institutes of Health (NIH) Institutional Review Board approved protocol (no. 99-CC-0168) (https://clinicalstudies.info.nih.gov/protocoldetails.aspx?id=99-CC-0168&&query=). Peripheral blood mononuclear cells from three healthy blood donors—Donor 1 (male, 45-year-old, Native American), Donor 2 (female, 47-year-old, White), and Donor 3 (female, 53-year-old, White)—were isolated over Ficoll and cryopreserved. Naive CD19^+^ human B cells were isolated with EasySep Human Naive B Cell Isolation Kit according to the manufacture’s instructions (Stemcell Technologies). Isolated B cells were grown in IMDM media supplemented with 10% FBS (Gemini), 50 μM of beta-mercaptoethanol, and 1% Penicillin-Streptomycin (Thermo Fisher Scientific). Cells were activated with 100 ng/mL MEGACD40L (Enzo Life Sciences), 1 μg/mL CpG oligodeoxynucleotide ODN 2006 (Invivogen), 50 ng/mL human IL-2, 50 ng/mL human IL-10, and 10 ng/mL human IL-15 (all interleukins from PeproTech). Every other day the medium was fully removed and cells diluted in fresh activation medium.

All cells were cultured at 37°C and 5% CO2 in a humidified incubator.

### METHOD DETAILS

#### CRISPR/Cas9 engineering of gene reporter cell lines

Gene reporter SU-DHL-4 cell lines were created by CRISPR/Cas9-mediated mO or GFP knock-in via homologous recombination using sgRNA that cut in proximity to the start codon for N-terminal or stop codon for C-terminal knock-in. The choice between N- and C-terminal was based on the presence of mRNA isoforms with different transcription initiation or termination sites in order to target most transcripts. Of note, SU-DHL-4 cells carry a t(14;18) translocation between BCL2 and the immunoglobulin heavy chain locus (*BCL2*:*IGH*).^95^ To distinguish between the two, we labeled the translocated BCL2 allele C-terminal and the functional IGH allele N-terminal. sgRNAs were selected using the sgRNA online tool (https://crispr.zhaopage.com) to maximize on-target efficiency and minimize off-target risk. sgRNA were cloned into px458-mOrange or px458-GFP (Addgene, 48138). Based on the selected sgRNA, targeting constructs were cloned using a 2-step ‘Stitch’ PCR approach. In the first step, three separate PCR reactions were set up to amplify the 5′ homology arm (PCR 1), the fluorescent marker (mO or GFP) linked with a self-excising peptide sequence (P2A or T2A) in frame with the target gene (PCR 2), and the 3′ homology arm (PCR 3). Silent mutations were introduced into the targeting construct’s sgRNA sequence to prevent cutting by CRISPR/Cas9. PCR products were gel purified and mixed together. As products of PCR 1–3 contain matching overlapping sequences, in the second PCR step, they were combined together by overlapping (Stitch) PCR (Table S1 for sgRNA and primer information). Final PCR products were gel purified and cloned into Zero Blunt TOPO (Thermo Fisher Scientific). One million SU-DHL-4 cells were transfected with targeting construct (1 μg) and sgRNA vector (1 μg) using a 4D-Nucleofector (Program CM150) with SF Cell Line 4D-Nucleofector X Kit (Lonza). After one week of recovery, mO^+^ or GFP^+^ cells were sorted on a BD FACS Aria or Fusion cell sorter. Single cell clones were analyzed for mO or GFP expression over a period of 2–3 weeks and clones with a uniform and stable expression were expanded. Clones were genotyped and heterozygous knock-in clones selected for CRISPR screening, except for BCL11A for which we only obtained bright enough homozygous clones.

#### sgRNA library amplification and virus production

The genome-wide sgRNA library Brunello^14^ was purchased from Addgene and amplified following the recommended instructions.^96^ Viral particles were produced by transfecting HEK293T cells with the amplified Brunello library, the packing plasmid psPAX2, and the envelope plasmid pVSV-G in the presence of Lipofectamine LTX and Plus reagent (Thermo Fisher Scientific). Viruses were harvested 48h and 72h after transfection. Collected supernatants were pooled, 0.45 μm sterile filtered, and concentrated by ultracentrifugation (Beckman XL-90 Ultracentrifuge, SW28 rotor, 25,000 rpm, 2 h, 4°C). Aliquots of concentrated viruses were snap-frozen and conserved at −80°C until subsequent use.

#### Genome-wide CRISPR/Cas9 regulator screens

SU-DHL-4 reporter cells were transduced with the Brunello library. For each screen, 6 replicates with 15 million cells (90 million in total) were transduced at a density of 5 million cells/mL in a 6 well plate. The amount of virus was adjusted according to the viral titer to obtain a multiplicity of infection (MOI) of 0.3. Cells were transduced by spinfection (1,460g for 90 min and 30°C) in the presence of 6 μg/mL polybrene. Infected cells were puromycin selected 24h post infection at a final concentration of 4 μg/mL. On day 3 post infection, the MOI was verified and cells were split in medium containing 200 ng/mL doxycycline. Cells were split every other day with fresh doxycycline containing medium. On day 10 post infection, for each of the six replicates 60 million cells were sorted using BD FACS Aria or Fusion cell sorters based on reporter fluorescence to capture the bottom and top 5% expressing cells. On average, for each screen 286 million cells were sorted yielding ~9 million low or high expressing cells (Table S2). For the three mTORC1 inhibited CRISPR screens in *BTG2*, *CD79B* and *OCAB* reporter cell lines, 5 nM rapamycin was added on day 8 and cells were sorted on day 10 as for the other screens. Genomic DNA from sorted cells was extracted using DNeasy Blood and Tissue kit (Qiagen) according to the manufacturer’s instructions. Libraries suitable for next-generation sequencing were prepared using staggered GeCKO readout primers following the recommended instructions.^87^ Deep sequencing was performed on NextSeq550 and NovaSeq6000 (Illumina) using 75bp single-end reads.

#### Individual regulator hit validation

*OCAB* regulators were validated by individually transducing the sgRNA detected with the lowest RRA score from the Brunello library. For this, selected sgRNAs and a non-targeting sgRNA from the Brunello library were cloned into lentiGuide-puro (Addgene). Viral particles were produced in 24 well plates by transfecting HEK293T cells with the sgRNA containing lentiGuide-puro, the packing plasmid psPAX2, and the envelope plasmid pVSV-G in the presence of Lipofectamine LTX and Plus reagent (Thermo Fisher Scientific). Viruses were harvested 48h after transfection and used to infect *mO-OCAB* SU-DHL-4 cells by spinfection (1,460g for 90 min and 30°C) in the presence of polybrene (6 μg/mL). One day post infection, infected cells were selected with puromycin at a final concentration of 4 μg/mL. Three days post infection, 200 ng/mL doxycycline was added and cells were split every other day with fresh doxycycline containing medium. mO-OCAB reporter expression was analyzed by flow cytometry 10 days post infection and mean reporter intensity was calculated relative to non-targeting sgRNA. MFI changes of >5% (reduction in case of activators and increase for repressors) were considered true positives and accordingly changes of <5% false positives.

#### Gene regulatory network

Based on the identified regulators in each CRISPR screen (Tables S3A and S3B), Cytoscape was used to create a gene regulatory network containing all 4,440 detected regulators and a total of 17,638 regulatory connections (6,487 activating and 11,151 repressing). For this, the lists of identified activators and repressors were combined in a single table together with their targets, RRA scores, and regulatory direction (0 or 1 for repression or activation, respectively). A gene regulatory network was created in Cytoscape by selecting the regulator column as source node, the screen as target node, and the RRA score and regulatory direction as edge attributes.

#### Hierarchical regulator clustering

Hierarchical clustering based on regulator overlap between the 47 CRISPR screens was performed separately for activator or repressors to determine regulator overlaps. We noticed two groups of genes that were frequently regulated by identical activators (group 1) or repressors (group 2). Based on the automated clustering, CRISPR screens were arranged to best separate group 1 and group 2 genes by simultaneously taking activator and repressor overlaps into account.

#### STRING analysis

The STRING App in Cytoscape was used to interrogate the biological function of regulators identified in the CRISPR screens. Regulators were queried in a full STRING network comprising physical and functional interactions with a confidence score cutoff of 0.4 and without adding additional interactors. Activators (*n* = 2,665) and repressors (*n* = 2,740) found in the STRING database were analyzed separately. Markov clustering was used to cluster regulators by biological function and in total 424 activator and 403 repressor clusters were obtained (Tables S4A and S4B). STRING’s functional enrichment tool was used to determine the biological function of each cluster. Differentially expressed genes determined by RNA-seq in rapamycin-treated SU-DHL-4 cells were analyzed similarly. Significantly down- and up-regulated genes were analyzed separately by STRING in Cytoscape (Tables S6A and S6B).

#### Flow cytometric analysis

To determine the effect of genetic or pharmaceutical perturbations on gene expression, reporter fluorescence in the 47 SU-DHL-4 reporter cell lines was measured by flow cytometry. Reporter lines expressed either GFP or mO and were run in 96 well plates on a BD Canto or BD Fortessa flow cytometer. Data was analyzed on FlowJo. Inhibitor treated cells were analyzed 2 days post-treatment and mean reporter intensity was calculated relative to DMSO. For genetic perturbations, sgRNA transduced cells were analyzed on day 10 post-infection and mean reporter intensity was calculated relative to non-targeting sgRNA. For experiments that used combined treatments (rapamycin + GSK3i or rapamycin + GSK3-targeting sgRNAs) mean reporter intensity was calculated relative to single treatment (GSK3i or GSK3-targeting sgRNAs) to determine the effect of rapamycin in those backgrounds. For G1 cell cycle analysis, cells were stained with Hoechst 33342 (Molecular Probes) at a final concentration of 5 μg/mL. All experiments were performed in triplicate and mean results or representative replicates are shown.

#### Inhibitor treatments

SU-DHL-4 or primary B cells were seeded at 0.2 million cells/mL and treated with DMSO or inhibitors. Inhibitors were purchased from MedChemExpress or Selleck Chemicals and dissolved in DMSO. Inhibitors were titrated and used at the following concentrations: rapamycin (5 nM), MK2206 (250 nM), AS1842856 (50 nM), LY2090314 (2 nM), CHIR99021 (1 μM). After two days of treatment, cells were analyzed by flow cytometry or harvested for RNA-seq. All experiments were performed in triplicate and mean results are shown.

#### Kinase inhibition assay

*In vitro* kinase HotSpot assays was performed against GSK3A and GSK3B in a 10-dose IC50 triplicate setup by Reaction Biology.

#### RNA sequencing (RNA-seq)

Cells for RNA-seq analysis were either unperturbed, transduced with sgRNA, or treated with inhibitors as described above. Cells were split every other day and seeded at 200,000 cells/mL the day before harvest. Inhibitor treated cells were harvested on day 2 post-treatment and sgRNA transduced cells on day 10 post-infection. On the day of harvest, half a million cells were pelleted and lysed in 100 μL of the Ambion RNAqueous lysis solution. RNA was extracted and treated with DNase according to the RNAqueous-Micro Total RNA Isolation Kit protocol (Thermo Fisher Scientific). RNA-seq libraries were prepared from 500 ng of RNA using NEBNext Poly(A) mRNA Magnetic Isolation and NEBNext Ultra II Directional RNA Library Prep Kit for Illumina (New England Biolabs). Single 50 cycles of sequencing data were acquired on HiSeq3000, NovaSeq6000 and NovaSeqXplus (illumina). All experiments were performed in triplicate.

#### Chromatin immunoprecipitation sequencing (ChIP-seq)

Cells for ChIP-seq analysis were either unperturbed or transduced with sgRNA for 10 days before harvesting. The day before harvest, cells were diluted to 200,000 cells/mL. Cultured cells were fixed at room temperature for 10 min with 1% formaldehyde (Sigma) and the reaction was quenched with 125 mM glycine (Sigma). Fixed cells were washed with PBS, snap-frozen, and stored at −80°C until further processing. For ChIP, 20 million cells were resuspended in 1 mL of RIPA buffer (10 mM Tris pH 7.6, 1 mM EDTA, 0.1% SDS, 0.1% sodium deoxycholate, 1% Triton X-100, and freshly added Complete Mini EDTA free proteinase inhibitor (Roche)). Chromatin was sheared by sonication on ice using a Branson Sonifier with an amplitude of 35% and 12 cycles of 20 s sonication followed by 30 s of pause. Supernatant was precleared with 20 μL Dynabeads Protein A (Thermo Fisher Scientific) for 1 h at 4°C under rotation. Precleared chromatin was incubated with BRD4 (Abcam, ab128874) or FLI1 (Abcam, ab133485) antibody overnight at 4°C under rotation. The antibody-protein solution was then captured with 40 μL of Dynabeads Protein A for 1 h at 4°C under rotation. Antibody-bound beads were washed twice with RIPA buffer, twice with RIPA buffer containing 0.3 M NaCl, once with LiCl buffer (0.25M LiCl, 0.5% NP-40, 0.5% sodium deoxycholate), once with TE pH 8.0 containing 0.2% Triton X-100, and once with TE pH 8.0. Crosslinks were reversed by incubating the beads at 65°C for 4 h in the presence of 0.3% SDS and 1 mg/mL Proteinase K (Thermo Fisher Scientific). ChIP DNA was column purified with ChIP DNA Clean and Concentrator kit (Zymo research). Libraries were prepared using the Ovation Ultralow Library System V2. Single 50 cycles of sequencing data were acquired on NextSeq550 and NovaSeq6000 (illumina). All experiments were performed in duplicate and representative replicates are shown.

#### Proteomic profiling

SU-DHL-4 cells were treated with either rapamycin or DMSO for 1 or 2 days prior to proteomic analysis. For each replicate, a total of 6 million cells were harvested, washed twice with cold PBS, snap-frozen, and stored at −80°C until further processing. Comparative proteomic analysis was conducted by Poochon Scientific using the TMT-18plex labeling-based quantitative proteomics in combination with liquid chromatography-tandem mass spectrometry (LC-MS/MS). Both global protein and phospho-protein profiling were performed. In total, 6,612 proteins were quantitatively identified, including 2,109 phosphorylated proteins encompassing 6,660 phosphorylation sites. Proteins or phosphorylation sites with a fold change of ≥25% and a *p*-value <0.05 (*n* = 3) when comparing rapamycin-to DMSO-treated cells were considered significantly altered.

### QUANTIFICATION AND STATISTICAL ANALYSIS

#### CRISPR screen analysis

##### Generation of reference sequence

Template1 seq:

TCTTGTGGAAAGGACGAAACACCG.

Template2 seq:

GTTTTAGAGCTAGAAATAGCAAGTTAAAATAAGGCTAGTCCGTTATCAACTTGAAAAAGTGGCACCGAGTCGGTGCTTTTTTAAGCTTGGCGTAACTAGATCTTGAGACAAATGGCAGTATTCATCCACAATTTTAAAAGAAAAGGGGGGATTGGGGGGTACAGTGCAGGGGAAAGAATAGTAGA.

Primer seq.

**Table:** 

name	Stagger	Index
F01	t	AAGTAGAG
F02	at	ACACGATC
F03	gat	CGCGCGGT
F04	cgat	CATGATCG
F05	tcgat	CGTTACCA
F06	atcgat	TCCTTGGT
F07	gatcgat	AACGCATT
F08	cgatcgat	ACAGGTAT
F09	acgatcgat	AGGTAAGG
F10	t	AACAATGG
F11	at	ACTGTATC
F12	gat	AGGTCGCA

For each sgRNA sequence in Brunello library, add each primer stagger and index sequence, template1 sequence in front of sgRNA sequence and add template2 sequence at the end of sgRNA sequence. So total 929292 sequences (12*77441) are generated for 77441 sgRNA Brunello library and stored in one fasta file. The name of each sequence was made with combination of primer name, targeted gene name and sgRNA name. This enables to mix up to 12 libraries in one sequencing lane. Stagger sequence is used to give enough complexity in sequencing due to the common template sequence. Using bowtie/1.1.2 bowtie-build function,^89^ we built an artificial sgRNA genome index for mapping.

##### Alignment and demultiplexing

We mapped reads into the artificial sgRNA genome using bowtie//1.1.2 with -S -m 1 -p 4 -a –best –strata -n 2 -L 80 options. Using samtools, aligned reads are selected, sorted and indexed. Table were obtained through samtools idxstats function to show the number of reads matched to each sequence. By using custom script, we extract the sample, gene name, sgRNA name, count and fraction from the table.

##### MAGeCK run and extraction and filtering of outputs

For a statistical analysis, we used MAGeCK program^15^ by providing the low 5% replicates as test set and the high 5% replicates as control set with control sgRNA list from Brunello library. We selected candidates with 10^−3^ RRA score cutoff from the output of MAGeCK program and removed hits located between centromere and targeted genes as false positives. We also removed hits on transcriptionally inactive genes of which rpkm is less than 0.1. Centromere information is extracted from UCSC genome browser gap tables in human genome. Pos.score is used for score of low population and neg.score is used for high population.

**Table:** 

Primer	All others	BLK_IGK_2	MEF2B_2
F01	rep1 low	BLK rep7 low	
F02	rep1 high	BLK rep7 high	
F03	rep2 low	BLK rep8 low	
F04	rep2 high	BLK rep8 high	
F05	rep3 low	BLK rep9 low	
F06	rep3 high	BLK rep9 high	
F07	rep4 low	IGK rep7 low	rep7 low
F08	rep4 high	IGK rep7 high	rep7 high
F09	rep5 low	IGK rep8 low	rep8 low
F10	rep5 high	IGK rep8 high	rep8 high
F11	rep6 low	IGK rep9 low	rep9 low
F12	rep6 high	IGK rep9 high	rep9 high

#### RNA-seq analysis

Reads were aligned to the human genome (hg19) with using gsnap,^91^ without detecting splice junctions *de novo* (–novelsplicing = 0). Existing splice junctions from the RefSeq annotation were taken into account (–use-splicing = /path/to/hg19.splices.iit). The output files were filtered to remove unaligned reads and any alignments with a mapping quality less than 20. Reads were then mapped to RefSeq genes using “htseq-count” with the “-m intersection-nonempty” option.^92^ The basemean, fold-change (FC) and adjusted *p*-values (Benjamini-Hochberg method) for the differentially expressed gene analysis were calculated using the R package DESeq2.^90^ Genes with more than 100 basemean, greater than 1.5 FC and less than 0.01 adjusted *p*-values were selected as the significantly differentially expressed genes. For pathway enrichment analysis, we used ReactomePA R package. Gene set enrichment analysis on DEGs was performed using WebGestalt selecting gene ontology as a functional database with non-redundant biological processes. GSEA calculates false discovery rates through permutation testing.

#### ChIP-seq analysis

Sequence reads were aligned to the human genome (hg19) using bowtie/2 with flags -S -m 1 -a –best –strata -n 2, and aligned reads were selected with samtools view -S -b -F4 and sorted. Using picard, we removed duplication, then we extended the reads into the estimated fragment sizes by MaSC.^94^ Density tracks were generated using custom software based on the samtools library to count the number of reads in 100 bp windows normalized to window size and library size to obtain densities in units of reads per kb region per million mapped reads (rpkm) across the genome and converted to bigWig with bedGraphToBigWig (UCSC utilities).

## Supplementary Material

1

2

3

5

6

7

8

9

10

11

12

Supplemental information can be found online at https://doi.org/10.1016/j.celrep.2025.116361.

## Figures and Tables

**Table:** KEY RESOURCES TABLE

REAGENT or RESOURCE	SOURCE	IDENTIFIER
Antibodies		
BRD4	Abcam	ab128874; RRID: AB_11145462
FLI1	Abcam	ab133485; RRID: AB_2722650
Bacterial and virus strains		
Human CRISPR Knockout Pooled Library (Brunello)	Addgene	73178
lentiGuide-puro	Addgene	52963
Chemicals, peptides, and recombinant proteins		
Advanced RPMI-1640 medium	Thermo Fisher Scientific	12633012
AS1842856	MedChemExpress	HY-100596
beta-mercaptoethanol	Thermo Fisher Scientific	21985–023
Complete Mini EDTA free proteinase inhibitor	Roche	05892791001
CpG oligodeozynucleotide ODN 2006	Invivogen	TLRL-2006-1
DMEM (Dulbecco’s Modified Eagle Medium)	Thermo Fisher Scientific	11960–044
DMSO	Sigma Aldrich	D2650-100ML
Doxycycline	Sigma Aldrich	D2630-5g
Dynabeads Protein A	Thermo Fisher Scientific	10002D
FBS (Fetal bovine serum)	Gemini - Bio Products	100–106
Formaldehyde solution	Sigma Aldrich	F1635-25mL
GlutaMAX	Thermo Fisher Scientific	35050–061
Glycine	Sigma Aldrich	G7403-250G
Hoechst 33342	Molecular Probes	H-3570
Human IL-10	PeproTech	200-10-10UG
Human IL-15	PeproTech	200-15-10UG
Human IL-2	PeproTech	200-02-10UG
IMDM (Iscove’s Modified Dulbecco’s Medium) + GlutaMAX	Thermo Fisher Scientific	31980–030
Lipofectamine LTX with PLUS reagent	Thermo Fisher Scientific	15338100
LY2090314	MedChemExpress	HY-16294
MEGACD40L	Enzo Life Sciences	ALX-522-110-C010
MK2206	Selleck Chemicals	S1078
Opti-MEM I Reduced Serum Medium	Thermo Fisher Scientific	31985–070
PBS (Phosphate buffered saline)	Thermo Fisher Scientific	10010–023
Penicillin-Streptomycin	Thermo Fisher Scientific	15140122
Polybrene	Sigma Aldrich	107689-10G
Proteinase K	Thermo Fisher Scientific	26160
Puromycin	Thermo Fisher Scientific	A11138-03
Rapamycin/Sirolimus	Selleck Chemicals	S1039
Sodium Pyruvate	Thermo Fisher Scientific	11360–070
Stemolecule CHIR99021	Stemgent	04-0004-02
Critical commercial assays		
ChIP DNA Clean & Concentrator Kit	Zymo Research	D5205
DNA Clean & Concentrator Kit	Zymo Research	D4013
DNeasy Blood & Tissue Kit	QIAGEN	69506
EasySep Human Naive B Cell Isolation Kit	Stemcell Technologies	17254
*In vitro* kinase HotSpot assays	Reaction Biology	N/A
NEBNext Poly(A) mRNA Magnetic Isolation Module	New England Biolabs	E7490L
NEBNext Ultra II Directional RNA Library Prep Kit for Illumina	New England Biolabs	E7760
Ovation Ultralow Library System V2	Tecan Genomics	0344NB-A01
Proteomic profiling	Poochon Scientific	P303
Q5 High-Fidelity DNA polymerase	New England Biolabs	M0491L
RNAqueous-Micro Total RNA Isolation Kit	Thermo Fisher Scientific	AM1931
SF Cell Line 4D-Nucleofector X Kit	Lonza	V4XC-2032
ZeroBlunt TOPO	Thermo Fisher Scientific	450245
ZR Plasmid Miniprep	Zymo Research	D4015
Zymoclean Gel DNA Recovery Kit	Zymo Research	D4002
Deposited data		
NGS Deep-sequencing raw data	This paper	GEO: GSE290879
Mass spectrometry proteomics data	This paper	PRIDE: PXD067812
B-LEARN source code	This paper	Zenodo: https://doi.org/10.5281/zenodo.16120942
Experimental models: Cell lines		
HEK293T	ATCC	N/A
SU-DHL-4 with doxycycline-inducible Cas9	Phelan et al.^13^	N/A
SU-DHL-4_AKT2-T2A-GFP	This paper	N/A
SU-DHL-4_BACH2-T2A-GFP	This paper	N/A
SU-DHL-4_mOrange-P2A-BCL11A	This paper	N/A
SU-DHL-4_BCL2-T2A-GFP	This paper	N/A
SU-DHL-4_BCL2L1-T2A-GFP	This paper	N/A
SU-DHL-4_BCL6-T2A-mOrange	This paper	N/A
SU-DHL-4_BLK-T2A-mOrange	This paper	N/A
SU-DHL-4_BLNK-T2A-GFP	This paper	N/A
SU-DHL-4_BTG2-T2A-mOrange	This paper	N/A
SU-DHL-4_BTK-T2A-GFP	This paper	N/A
SU-DHL-4_GFP-P2A-CD27	This paper	N/A
SU-DHL-4_CD79A-T2A-GFP	This paper	N/A
SU-DHL-4_CD79B-T2A-GFP	This paper	N/A
SU-DHL-4_CD81-T2A-GFP	This paper	N/A
SU-DHL-4_CSK-T2A-GFP	This paper	N/A
SU-DHL-4_E2F2-T2A-GFP	This paper	N/A
SU-DHL-4_HDAC1-T2A-GFP	This paper	N/A
SU-DHL-4_ID3-T2A-GFP	This paper	N/A
SU-DHL-4_GFP-P2A-IgH	This paper	N/A
SU-DHL-4_IgK-T2A-mOrange	This paper	N/A
SU-DHL-4_IKZF1-T2A-mOrange	This paper	N/A
SU-DHL-4_IRF8-T2A-GFP	This paper	N/A
SU-DHL-4_LYN-T2A-GFP	This paper	N/A
SU-DHL-4_GFP-P2A-MAP3K7	This paper	N/A
SU-DHL-4_GFP-P2A-MCL1	This paper	N/A
SU-DHL-4_mOrange-P2A-MEF2B	This paper	N/A
SU-DHL-4_GFP-P2A-MS4A1	This paper	N/A
SU-DHL-4_GFP-P2A-MSH2	This paper	N/A
SU-DHL-4_MSH6-T2A-GFP	This paper	N/A
SU-DHL-4_MYC-T2A-GFP	This paper	N/A
SU-DHL-4_GFP-P2A-NCOA3	This paper	N/A
SU-DHL-4_GFP-P2A-PARP1	This paper	N/A
SU-DHL-4_PAX5-T2A-mOrange	This paper	N/A
SU-DHL-4_PIK3AP1-T2A-GFP	This paper	N/A
SU-DHL-4_mOrange-P2A-PLCG2	This paper	N/A
SU-DHL-4_mOrange-P2A-POU2AF1	This paper	N/A
SU-DHL-4_GFP-P2A-POU2F2	This paper	N/A
SU-DHL-4_mOrange-P2A-PTEN	This paper	N/A
SU-DHL-4_mOrange-P2A-PTPRC	This paper	N/A
SU-DHL-4_GFP-P2A-RB1	This paper	N/A
SU-DHL-4_GFP-P2A-REL	This paper	N/A
SU-DHL-4_SPI1-T2A-GFP	This paper	N/A
SU-DHL-4_SPIB-T2A-GFP	This paper	N/A
SU-DHL-4_SYK-T2A-GFP	This paper	N/A
SU-DHL-4_TCF3-T2A-GFP	This paper	N/A
SU-DHL-4_GFP-P2A-TNIP1	This paper	N/A
SU-DHL-4_GFP-P2A-TP53	This paper	N/A
Experimental models: Organisms/strains		
Human naive B cells	This paper	NIH blood bank
Oligonucleotides		
Cloning primers and sgRNA (for details see Table S1)	This paper	N/A
GeCKO readout primers	Shalem et al.^87^	N/A
sgRNA non-targeting control: AGCACGTAATGTCCGTGGAT	Brunello sgRNA library	Addgene, 73178
sgRNA targeting AMRBA1: CCATAATATCTATATTACGG	Brunello sgRNA library	Addgene, 73178
sgRNA targeting FLI1: ACTGTGTAAAATGAACAAGG	Brunello sgRNA library	Addgene, 73178
sgRNA targeting FOXO1: ACAGGTTGCCCCACGCGTTG	Brunello sgRNA library	Addgene, 73178
sgRNA targeting GSK3A: TCAGCCTCACAATATTGCAG	Brunello sgRNA library	Addgene, 73178
sgRNA targeting GSK3B: GTGGCTCCAAAGATCAACTC	Brunello sgRNA library	Addgene, 73178
sgRNA targeting IRF8: ATGGCTCGGAAATGTCCAGT	Brunello sgRNA library	Addgene, 73178
sgRNA targeting KLC2: CCGGCCTCAGGTGCAACCAG	Brunello sgRNA library	Addgene, 73178
sgRNA targeting SPEN: GATATTACCCGGGAGGTACG	Brunello sgRNA library	Addgene, 73178
Recombinant DNA		
pSpCas9(BB)-2A-GFP (PX458)	Addgene	48138
pSpCas9(BB)-2A-mOrange (PX458)	This study	N/A
psPAX2	Addgene	12260
pVSVg	Javier Di Noia	N/A
Software and algorithms		
B-LEARN	This paper	https://niams.shinyapps.io/B-LEARN/
Bedtools	Quinlan and Hall^88^	https://bedtools.readthedocs.io/en/latest/#
Bowtie and Bowtie2	Langmead et al.^89^	bowtie2 v2.1.0 http://bowtie-bio.sourceforge.net/index.shtml
Cytoscape	Cytoscape Consortium	https://cytoscape.org
DESeq2	Anders and Huber^90^	http://bioconductor.org/packages/release/bioc/html/DESeq2.html
FlowJo	Becton, Dickinson & Company	https://flowjo.com
Gsnap 2013-05-09	Wu and Nacu^91^	http://research-pub.gene.com/gmap/
Htseq	Anders et al.^92^	https://htseq.readthedocs.io/en/release_0.11.1/
MACS 2.1.0	Zhang et al.^93^	https://github.com/taoliu/MACS
MAGeCK	Li et al.^15^	https://sourceforge.net/projects/mageck/
MaSC	Ramachandran et al.^94^	https://drive.google.com/file/d/1T9qwKj9BUDhwJ4apCNu6kg2ohi3RiBbA/view
Picard Tools	Broad Institute	http://picard.sourceforge.net
WEB-based Gene SeT AnaLysis Toolkit	WebGestalt	https://www.webgestalt.org

## Highlights

- 47 CRISPR screens map a gene regulatory network with 4k regulators and 17k connections
- mTORC1 is a global regulator of B cell transcriptional activation and repression
- GSK3 counteracts mTORC1 in the regulation of cell cycle and immune response genes
- Immune response genes are regulated via modular, pathway-driven gene circuits
