# Supplementary material for: Biphasic control of the B cell transcriptome by mTORC1 and GSK3
Source: Cell Rep. Author manuscript; Available in PMC 2025 Nov 7. (PMC12594101; doi:10.1016/j.celrep.2025.116361)
Supplement: 1 [file NIHMS2120106-supplement-1.pdf]

Cell Reports, Volume *44*

Supplemental information

Biphasic control of the B cell transcriptome by mTORC1 and GSK3

Jens Kalchschmidt, Tomoya Kanno, Solji Park, Wendy D. Dubois, Yongbing Zhao, Pawel Trzaskoma, Craig J. Thomas, Louis M. Staudt, John J. O'Shea, Seolkyoung Jung, and Rafael Casellas

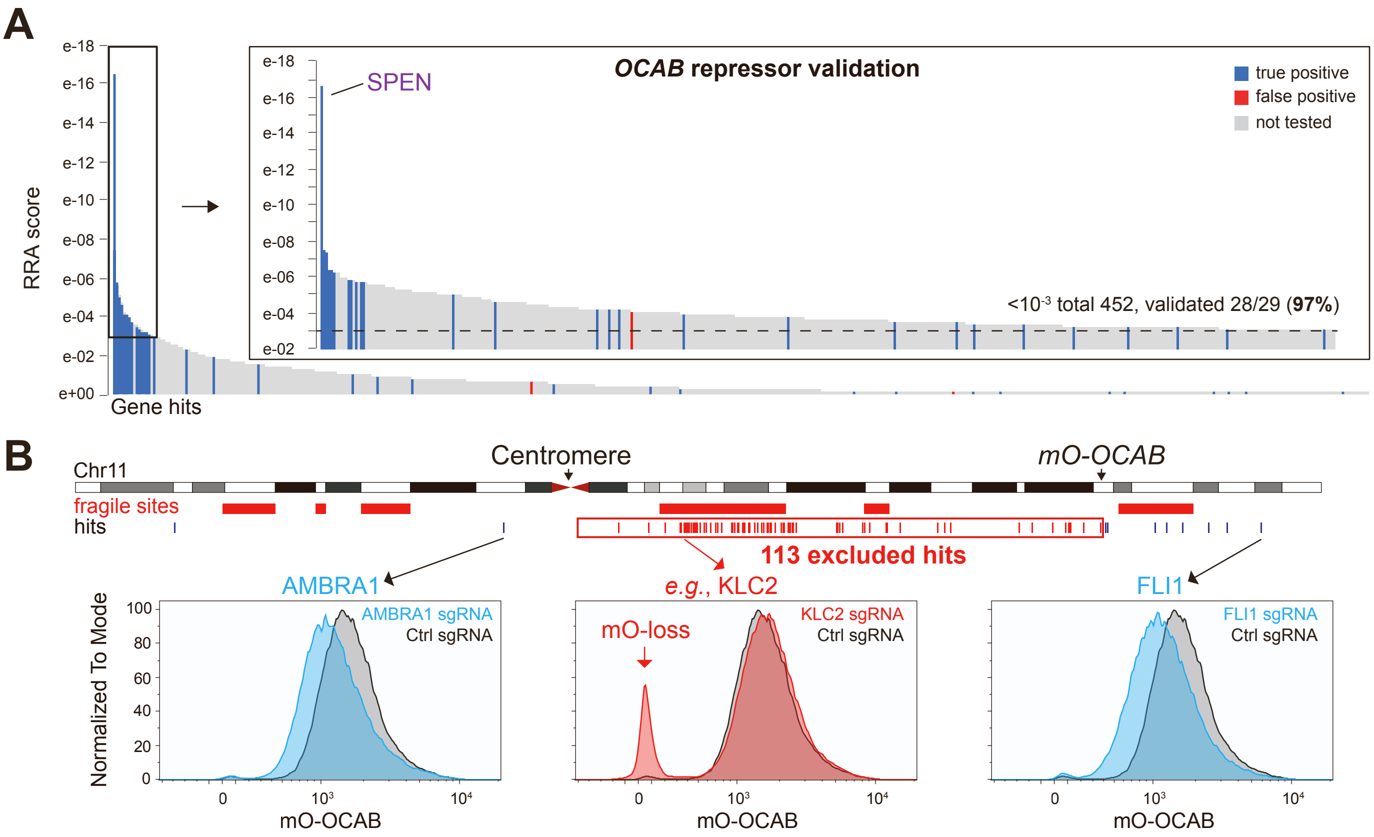

Figure S1. Validation of OCAB CRISPR/Cas 9 screen results, Related to Figure 1.

(**A**) Distribution of *OCAB* repressors ranked by RRA scores. Selected repressive regulators (marked by dark color) were tested again with specific targeting sgRNAs to ascertain assay reproducibility and 97% of examined factors with RRA scores below 10^{-3} were found to be reproducible.

(**B**) (Top) Distribution of chromosome fragile sites (from HumCFS) and *OCAB* activator hits across chromosome 11. Fragile sites and excluded hits are colored red. (Bottom) OCAB expression measured by flow cytometry after deleting *AMBRA1*, *KLC2*, or *FLI1* (each gene position denoted by arrow). A subset of cells that completely lost mO-OCAB signal was observed for *KLC2* deletion, indicative of a false positive hit due genome instability. Accordingly, hits that localized between the centromere and the mO-OCAB locus were excluded from downstream analyses (113 hits in red).

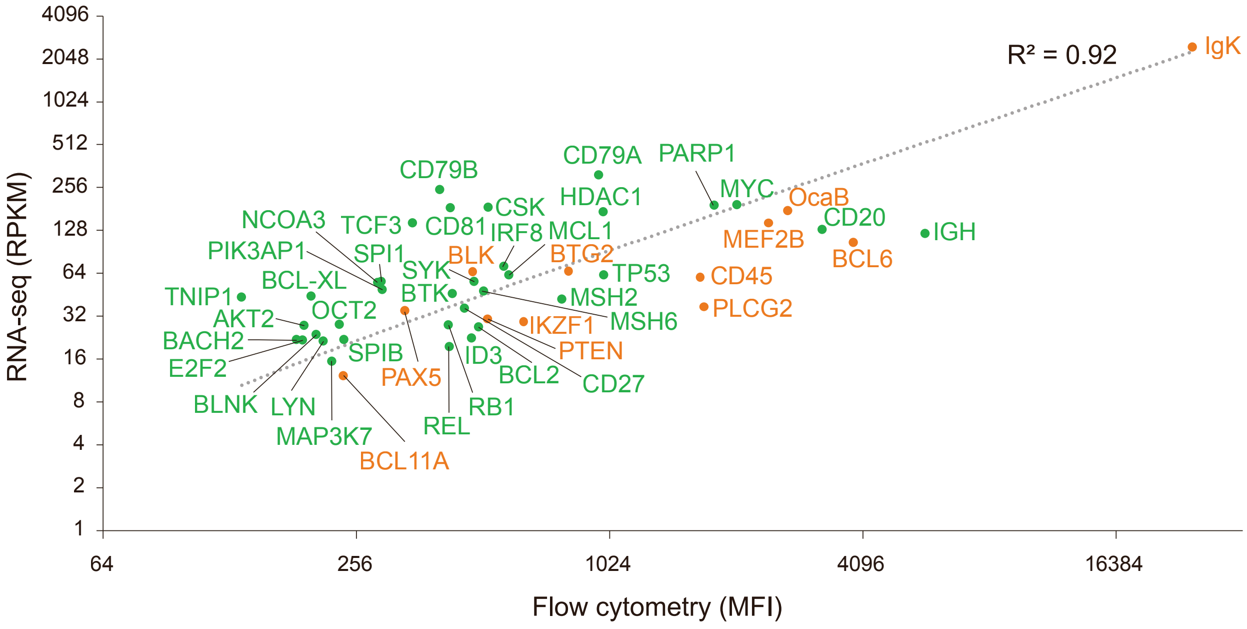

Figure S2. Validation of B cell gene reporter cell lines by flow cytometry and mRNA-seq, Related to Figure 2.

Scatterplot showing mRNA expression (RNA-seq) versus reporter mean fluorescence intensity (flow cytometry) for each of the 47 reporter cell lines. Genes labeled with mOrange are displayed in orange and genes labeled with GFP in green.

Figure S3

A

Activators

Top 25
across all 47 screens

Regulator	Screens detected
OTUD5	36
FZR1	35
COPS8	35
AMBRA1	35
CTR9	32
KCTD5	32
ATXN7L3	29
LEO1	28
CCNF	24
RB1	23
HIRA	20
YY1	20
ZNF330	20
ATRIP	19
TAF6L	19
WDR61	18
GABPB1	18
DDX3X	18
TAF2	18
PPP2R2A	17
RQCD1	17
ZBTB8OS	17
PAF1	16
SMG8	16
TADA1	16

C

Repressors

Top 25
by CRISPR score

Regulator	Target	RRA score
SPEN	OCAB	2.8E-17
METTL3	CD20	1.1E-14
N6AMT1	CD79B	1.1E-14
CCAR1	PARP1	2.3E-14
TIPARP	NCOA3	3.9E-13
RBM15	RB1	4.0E-13
NFKBIA	BCL-XL	8.3E-13
TSC2	CD20	1.5E-12
METTL3	MCL1	1.8E-12
DAD1	MEF2B	2.4E-12
GLRX5	AKT2	5.0E-12
NELFCD	RB1	5.6E-12
GLRX5	NCOA3	9.2E-12
PPP1R8	IGH	2.3E-11
METTL14	RB1	4.1E-11
SLC25A1	CD79A	6.8E-11
KCMF1	SPIB	7.5E-11
LRP10	BCL-XL	7.9E-11
PPP4C	TCF3	8.5E-11
EBF1	LYN	8.7E-11
BTG2	LYN	9.0E-11
SPI1	ID3	9.1E-11
SOD2	IGH	1.1E-10
SPEN	IGH	1.2E-10
CDK6	CD81	1.3E-10

Top 25
across all 47 screens

Regulator	Screens detected
CDK6	44
CCND3	41
FAM96B	41
DNAJC9	40
C19orf52	39
SOD2	37
POLD2	37
C17orf70	37
HSCB	37
CCNA2	36
RTEL1	35
PRPF8	35
GLRX5	35
NFS1	34
ZNHIT2	34
TIMELESS	33
RPS8	33
NARFL	33
TIMM10	33
WDR77	32
SKP2	32
PPP1R7	32
RFC1	31
ATP6V1B2	31
DNA2	30

B

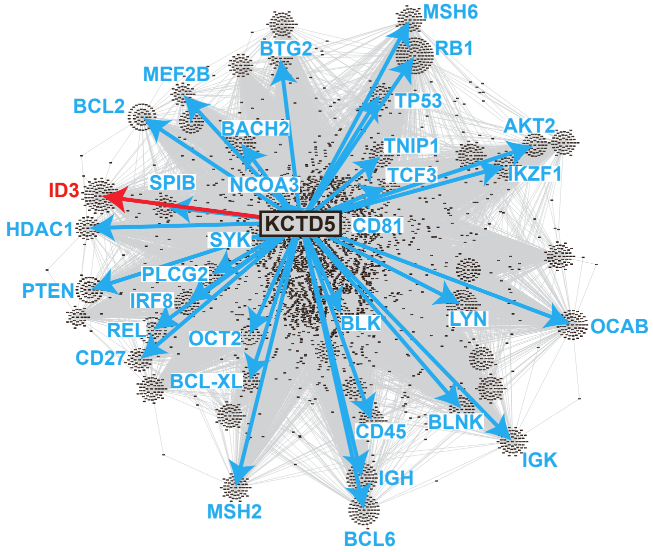

D

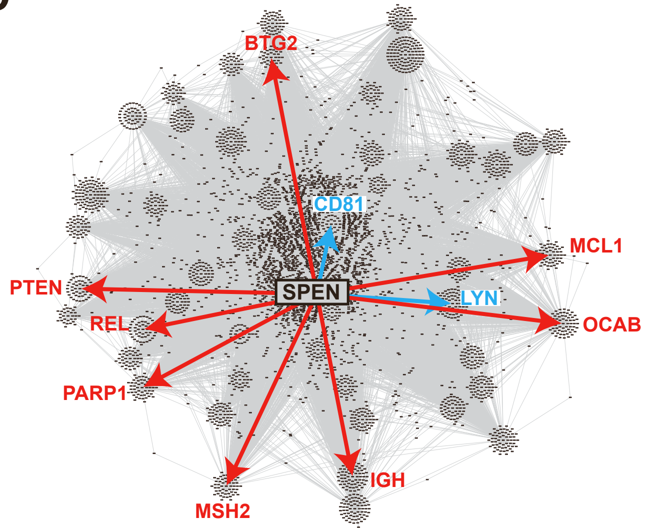

Figure S3. Top regulators that impact many targets or exhibit high RRA scores, Related to Figure 3.

(**A**) The top 25 most frequently detected activating regulators are listed based on the prevalence revealed in the 47 screens. Activator KCTD5 is highlighted in blue.

(**B**) Sub-network of regulator to target connections illustrating *KCTD5*'s dominant impact as an activator (blue: 32 genes) with limited action as repressor (red: 1 gene). The gray background represents the comprehensive gene regulatory network from all screens.

(**C**) List of the top 25 repressors ranked by RRA score (left) or ranked by the number of targets out of 47 screens (right). The repressor *SPEN* is highlighted in red.

(**D**) Sub-network of regulator to target connections for *SPEN*. *SPEN* acted dominantly as repressor (red: 8 genes) rather than an activator (blue: 2 genes).

Figure S4

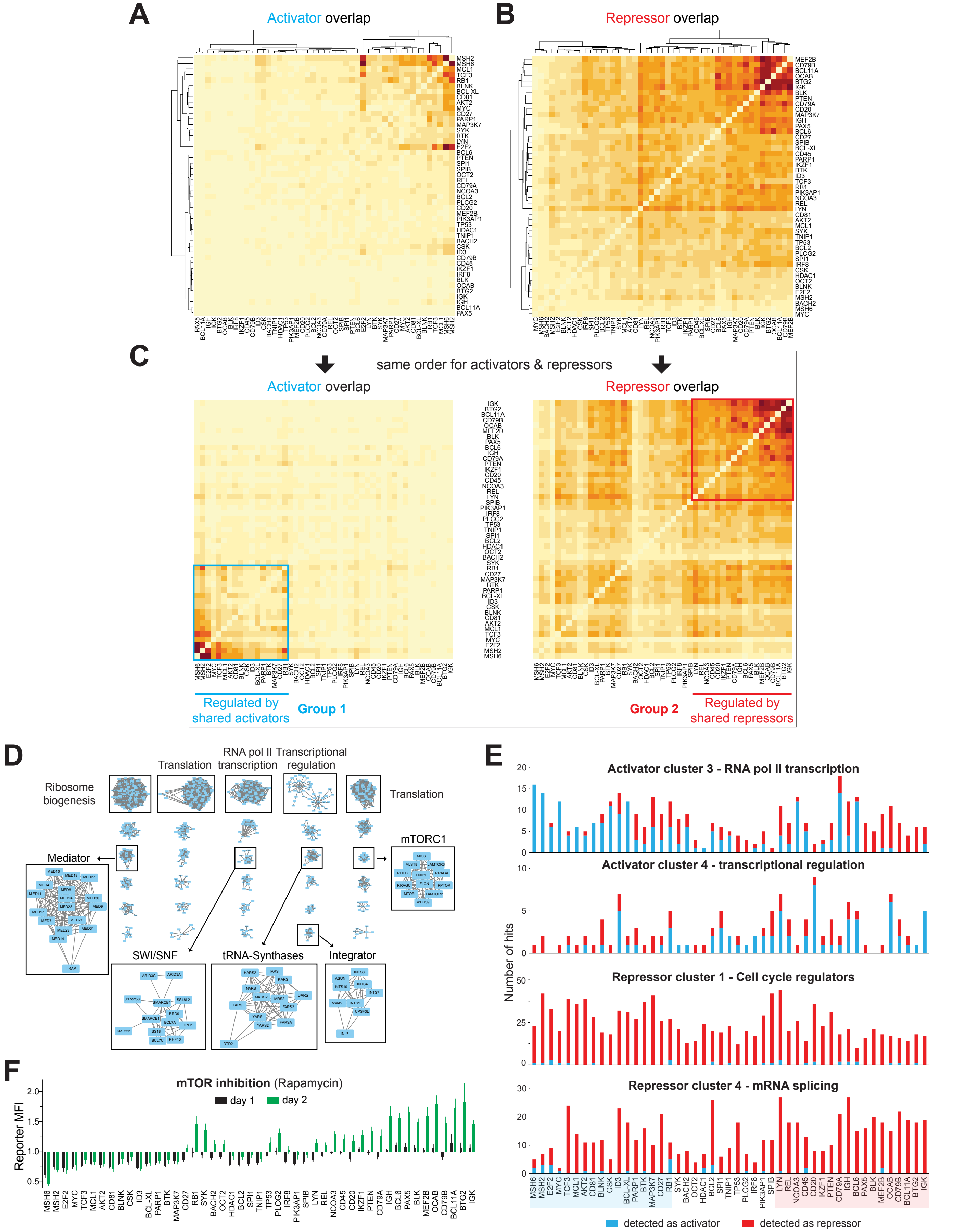

A
Activator overlap
B
Repressor overlap
C
same order for activators & repressors
Activator overlap
Repressor overlap
Regulated by shared activators
Group 1
Group 2
Regulated by shared repressors
D
Ribosome biogenesis
Translation
RNA pol II transcription
Transcriptional regulation
Translation
Mediator
mTORC1
SWI/SNF
tRNA-Synthases
Integrator
E
Activator cluster 3 - RNA pol II transcription
Activator cluster 4 - transcriptional regulation
Repressor cluster 1 - Cell cycle regulators
Repressor cluster 4 - mRNA splicing
Number of hits
detected as activator
detected as repressor
F
mTOR inhibition (Rapamycin)
day 1
day 2
Reporter MFI

Figure S4. Landscape of regulator network overlap and functional annotation of regulator modules, Related to Figure 5.

(**A**) Hierarchical clustering of activator overlaps amongst CRISPR screened genes.

(**B**) Hierarchical clustering of repressor overlaps of CRISPR screened genes.

(**C**) Based on panels (A+B), screened genes were rearranged in the same order for activator (left) and repressor (right) overlap. Genes regulated by shared activators or repressors are denoted by blue and red boxes, respectively.

(**D**) Top 30 activator clusters from STRING analysis.

(**E**) Bar graphs showing regulator counts per screened gene. Regulators belonging to indicated STRING clusters (Figure 5B) detected as activators or repressors were counted separately.

(**F**) Bar graph showing relative reporter MFI±SEM of rapamycin vs. DMSO treated cells at day 1 (black) or day 2 (green).

Figure S5

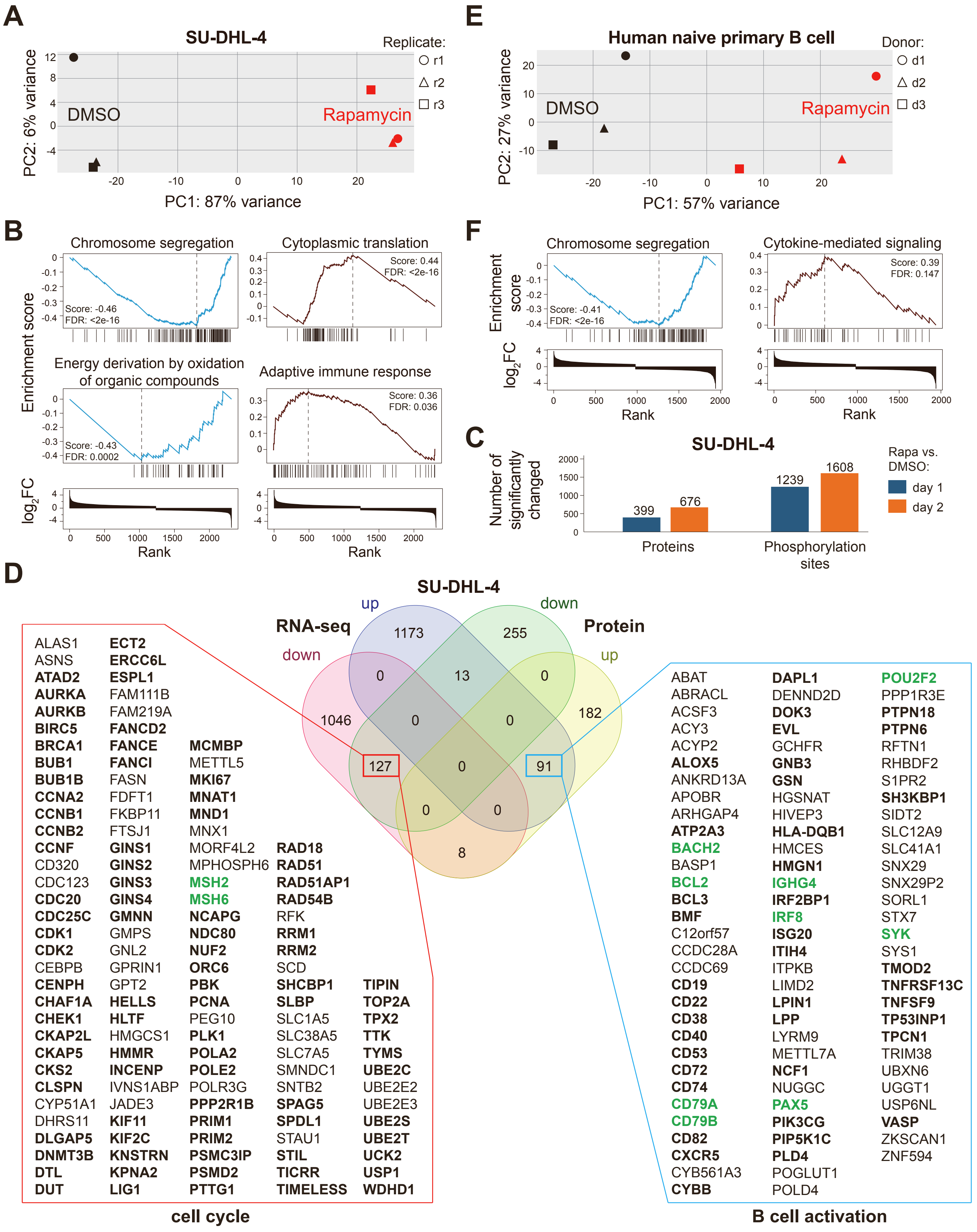

A
SU-DHL-4
Replicate:
r1
r2
r3
DMSO
Rapamycin
PC2: 6% variance
PC1: 87% variance
12
8
4
0
-4
-20
-10
0
10
20
E
Human naive primary B cell
Donor:
d1
d2
d3
DMSO
Rapamycin
PC2: 27% variance
PC1: 57% variance
20
10
0
-10
-20
-10
0
10
20
B
Enrichment score
Chromosome segregation
Score: -0.46
FDR: <2e-16
Cytoplasmic translation
Score: 0.44
FDR: <2e-16
Energy derivation by oxidation of organic compounds
Score: -0.43
FDR: 0.0002
Adaptive immune response
Score: 0.36
FDR: 0.036
log2FC
Rank
0
500
1000
1500
2000
F
Enrichment score
Chromosome segregation
Score: -0.41
FDR: <2e-16
Cytokine-mediated signaling
Score: 0.39
FDR: 0.147
log2FC
Rank
0
500
1000
1500
2000
C
SU-DHL-4
Number of significantly changed
Rapa vs. DMSO:
day 1
day 2
399
676
1239
1608
Proteins
Phosphorylation sites
0
500
1000
1500
2000
D
SU-DHL-4
RNA-seq
Protein
up
down
down
up
1173
255
0
13
0
1046
0
0
182
127
0
91
0
0
8
ALAS1
ASNS
ATAD2
AURKA
AURKB
BIRC5
BRCA1
BUB1
BUB1B
CCNA2
CCNB1
CCNB2
CCNF
CD320
CDC123
CDC20
CDC25C
CDK1
CDK2
CEBPB
CENPH
CHAF1A
CHEK1
CKAP2L
CKAP5
CKS2
CLSPN
CYP51A1
DHRS11
DLGAP5
DNMT3B
DTL
DUT
ECT2
ERCC6L
ESPL1
FAM111B
FAM219A
FANCD2
FANCE
FANCI
FASN
FDFT1
FKBP11
FTSJ1
GINS1
GINS2
GINS3
GINS4
GMNN
GMPS
GNL2
GPRIN1
GPT2
HELLS
HLTF
HMGCS1
HMMR
INCENP
IVNS1ABP
JADE3
KIF11
KIF2C
KNSTRN
KPNA2
LIG1
MCMBP
METTL5
MKI67
MNAT1
MND1
MNX1
MORF4L2
MPHOSPH6
MSH2
MSH6
NCAPG
NDC80
NUF2
ORC6
PBK
PCNA
PEG10
PLK1
POLA2
POLE2
POLR3G
PPP2R1B
PRIM1
PRIM2
PSMC3IP
PSMD2
PTTG1
RAD18
RAD51
RAD51AP1
RAD54B
RFK
RRM1
RRM2
SCD
SHCBP1
SLBP
SLC1A5
SLC38A5
SLC7A5
SMNDC1
SNTB2
SPAG5
SPDL1
STAU1
STIL
TICRR
TIMELESS
TIPIN
TOP2A
TPX2
TTK
TYMS
UBE2C
UBE2E2
UBE2E3
UBE2S
UBE2T
UCK2
USP1
WDHD1
cell cycle
ABAT
ABRACL
ACSF3
ACY3
ACYP2
ALOX5
ANKRD13A
APOBR
ARHGAP4
ATP2A3
BACH2
BASP1
BCL2
BCL3
BMF
C12orf57
CCDC28A
CCDC69
CD19
CD22
CD38
CD40
CD53
CD72
CD74
CD79A
CD79B
CD82
CXCR5
CYB561A3
CYBB
DAPL1
DENND2D
DOK3
EVL
GCHFR
GNB3
GSN
HGSNAT
HIVEP3
HLA-DQB1
HMCES
HMGN1
IGHG4
IRF2BP1
IRF8
ISG20
ITIH4
ITPKB
LIMD2
LPIN1
LPP
LYRM9
METTL7A
NCF1
NUGGC
PAX5
PIK3CG
PIP5K1C
PLD4
POGLUT1
POLD4
POU2F2
PPP1R3E
PTPN18
PTPN6
RFTN1
RHBDF2
S1PR2
SH3KBP1
SIDT2
SLC12A9
SLC41A1
SNX29
SNX29P2
SORL1
STX7
SYK
SYS1
TMOD2
TNFRSF13C
TNFSF9
TP53INP1
TPCN1
TRIM38
UBXN6
UGGT1
USP6NL
VASP
ZKSCAN1
ZNF594
B cell activation

Figure S5. Transcriptome and proteome analysis following prolonged rapamycin treatment, Related to Figure 6.

(**A**) RNA-seq PCA analysis depicting gene expression in 2-day rapamycin or DMSO treated SU-DHL-4 cells.

(**B**) Gene Set Enrichment Analysis (GSEA) for rapamycin downregulated (left) or upregulated (right) genes in SU-DHL-4 cells (related to Figure 6B). Gene sets used for plots are indicated on top of each panel. Scores and false discovery rates (FDR) based on permutation testing are displayed.

(**C**) Proteomic profiling of SU-DHL-4 cells treated with rapamycin for 1 or 2 days. Bar graph indicates the number of significantly altered proteins and phosphorylation sites identified at each time point.

(**D**) Venn diagram showing the overlap between significantly deregulated genes (RNA-seq, left) and proteins (proteomics, right) in SU-DHL-4 cells following 2-day rapamycin treatment. Lists highlight overlapping factors that are downregulated (left box) or upregulated (right box) at both the transcript and protein level. Genes included in our panel of 47 reporter cell lines are marked in green. Functional enrichment reveals that downregulated genes are associated with cell cycle regulation, while upregulated genes are linked to B cell activation (highlighted in bold).

(**E**) RNA-seq PCA analysis of primary B cells treated with 2-day rapamycin or DMSO.

(**F**) GSEA for rapamycin downregulated (left) or upregulated (right) genes in primary B cells (related to Figure 6E). Gene sets used for plots are indicated on top of each panel. Scores and false discovery rates (FDR) based on permutation testing are displayed.

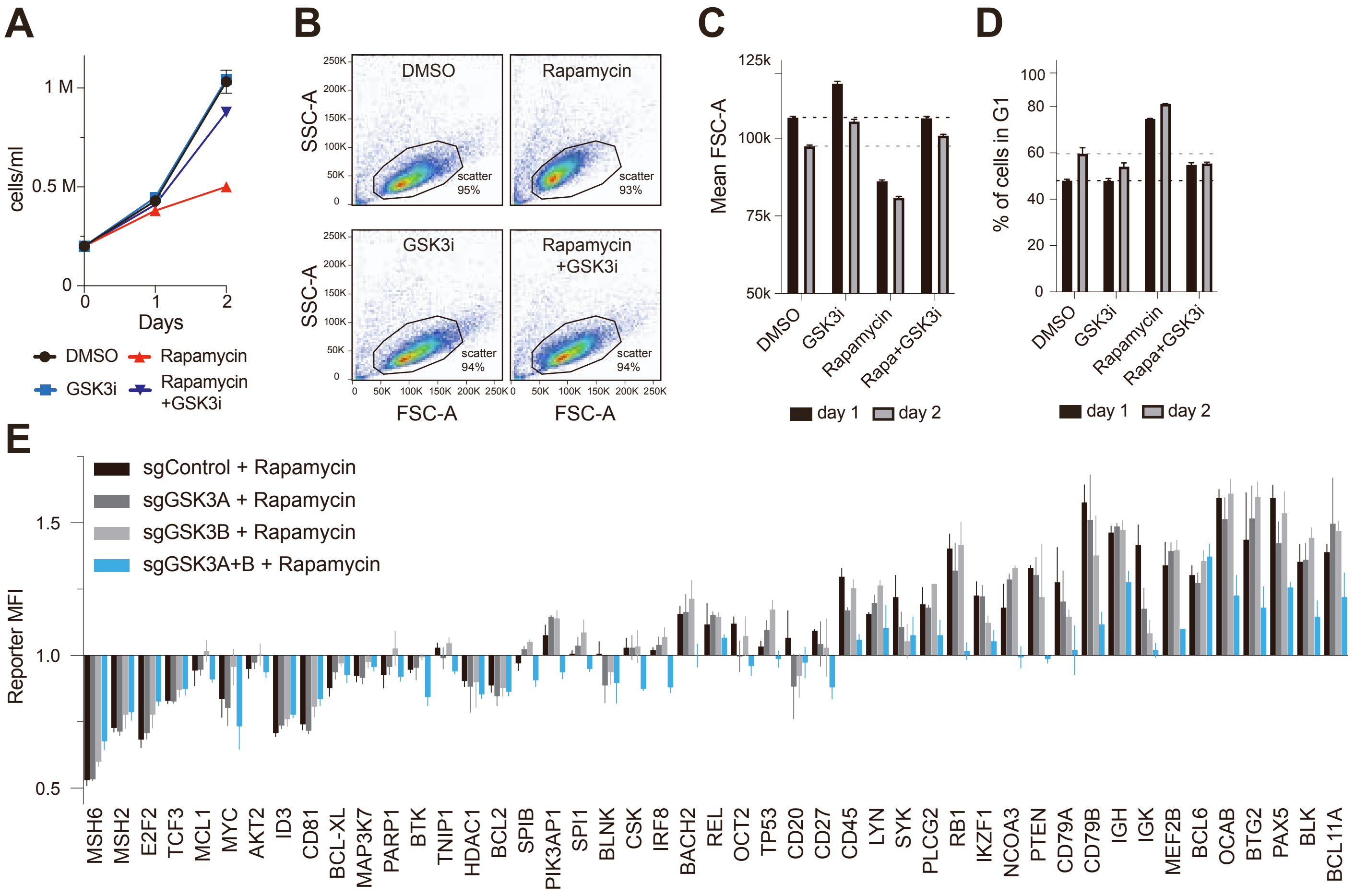

Figure S6. GSK3 inhibition counteracts rapamycin action, Related to Figure 7.

(**A**) Proliferation of DMSO or inhibitor treated SU-DHL-4 cells over 2 days.

(**B-D**) Cell size (B+C) and cell cycle (D) analyses of DMSO or inhibitor treated SU-DHL-4 cells.

(**E**) Bar graph showing relative reporter MFI of 2-day rapamycin treated control, GSK3A, GSK3B, or GSK3A+B depleted cells by sgRNA. MFI was calculated relative to DMSO treated cells of the same genotype. Data are represented as mean±SEM.
